# Industrialised fishing nations largely contribute to floating plastic pollution in the North Pacific subtropical gyre

**DOI:** 10.1038/s41598-022-16529-0

**Published:** 2022-09-01

**Authors:** Laurent Lebreton, Sarah-Jeanne Royer, Axel Peytavin, Wouter Jan Strietman, Ingeborg Smeding-Zuurendonk, Matthias Egger

**Affiliations:** 1 The Ocean Cleanup, Rotterdam, The Netherlands; 2 The Modelling House, Raglan, New Zealand; 3 Wageningen University & Research, Wageningen, The Netherlands; 4 Egger Research and Consulting, St Gallen, Switzerland

**Keywords:** Environmental sciences, Ocean sciences

## Abstract

The subtropical oceanic gyre in the North Pacific Ocean is currently covered with tens of thousands of tonnes of floating plastic debris, dispersed over millions of square kilometres. A large fraction is composed of fishing nets and ropes while the rest is mostly composed of hard plastic objects and fragments, sometimes carrying evidence on their origin. In 2019, an oceanographic mission conducted in the area, retrieved over 6000 hard plastic debris items > 5 cm. The debris was later sorted, counted, weighed, and analysed for evidence of origin and age. Our results, complemented with numerical model simulations and findings from a previous oceanographic mission, revealed that a majority of the floating material stems from fishing activities. While recent assessments for plastic inputs into the ocean point to coastal developing economies and rivers as major contributors into oceanic plastic pollution, here we show that most floating plastics in the North Pacific subtropical gyre can be traced back to five industrialised fishing nations, highlighting the important role the fishing industry plays in the solution to this global issue.

## Introduction

A large mass of plastics is currently floating and accumulating in the North Pacific subtropical gyre. This accumulation zone, referred to as the North Pacific Garbage Patch (NPGP), has been extensively documented^1–3^ and became a symbol of the impact of the widescale use of plastics and their discarding in the global ocean. However, it is well recognized that the mass of plastics accumulated at the surface of oceanic subtropical gyres like the NPGP represents only a small fraction of the global plastic emissions into the marine environment. With recent studies estimating up to several million tonnes of mismanaged plastic waste entering the world’s oceans from coastal cities^4^ and rivers worlwide^5–8^ every year, the larger part is believed to be predominantly accumulating on shorelines^9–13^ or on the seabed in proximity to landmasses^14–17^.

Litter monitoring programs and local cleanup efforts provide a useful tool to derive composition, abundance, sources and origins of plastic debris. At present, these programs are mostly focused on plastic debris collected from coastal environments^14,18–22^. There, the composition of accumulated plastic waste differs by location^22^. Negatively buoyant plastics are generally found closer to land-based sources while positively buoyant plastics dominate remote areas^11^. While maintaining buoyancy, these plastics can be transported at the sea surface and transported across oceans, influenced by a wide range of processes including currents, wind and waves^23^. The floating fraction of plastic pollution is highly problematic from an ecological perspective as positively buoyant plastic items represent a substantial vector for the transportation of invasive species^24–26^ and hence threaten biodiversity in other parts of the ocean^27,28^.

It is widely assumed that the majority of plastic debris in the ocean originates from land, but the contribution of oceanic sources was found greater in offshore regions^29^. Plastic drinking bottles, likely originating from passing shipping vessels, cover an uninhabited island of the South Atlantic Ocean^21^ while abandoned, lost or otherwise discarded fishing gear (ALDFG) accumulates on remote islands of the Pacific Ocean^30,31^. Oceanic sources such as inputs from fisheries have commonly been attributed about half of a million tonnes per year, but this estimate which has been repeatedly cited over the years, was misinterpreted from an initial study dating back to the 1970s^32^. Since then, no recent, more reliable estimate has been proposed. Although identified as a significant source of plastic debris in the ocean^33,34^, and representing a severe environmental^35^ and economic^36^ risk from entanglement, the spatial distribution and magnitude of ALDFG emissions remain very poorly understood. Modelling studies oriented to predict ALDFG pathways and accumulation are scarce but some regional studies have attempted to bridge this research gap^37,38^.

In offshore areas like in the NPGP, information on sources and origins of debris is often missing as most debris reported from expeditions are small fragments and fibres collected with surface net trawls^39^. A multi-vessels expedition in 2015 and aerial observations in 2016 over the NPGP revealed a significant fraction of larger debris such as fishing nets, ropes and other hard plastic objects > 5 cm, representing up to three-quarters of the accumulated floating plastic mass in the region^3^. It is trivial to attribute the accumulation of floating nets to fishing activities, but the fraction of hard plastics is likely a mix of different sources. Yet, while it is difficult to trace the country of origin for fishing nets or small plastic fragments, hard plastics > 5 cm can sometimes carry clues that lead to their age, as well as to their source and geographical origin.

**Figure 1:**
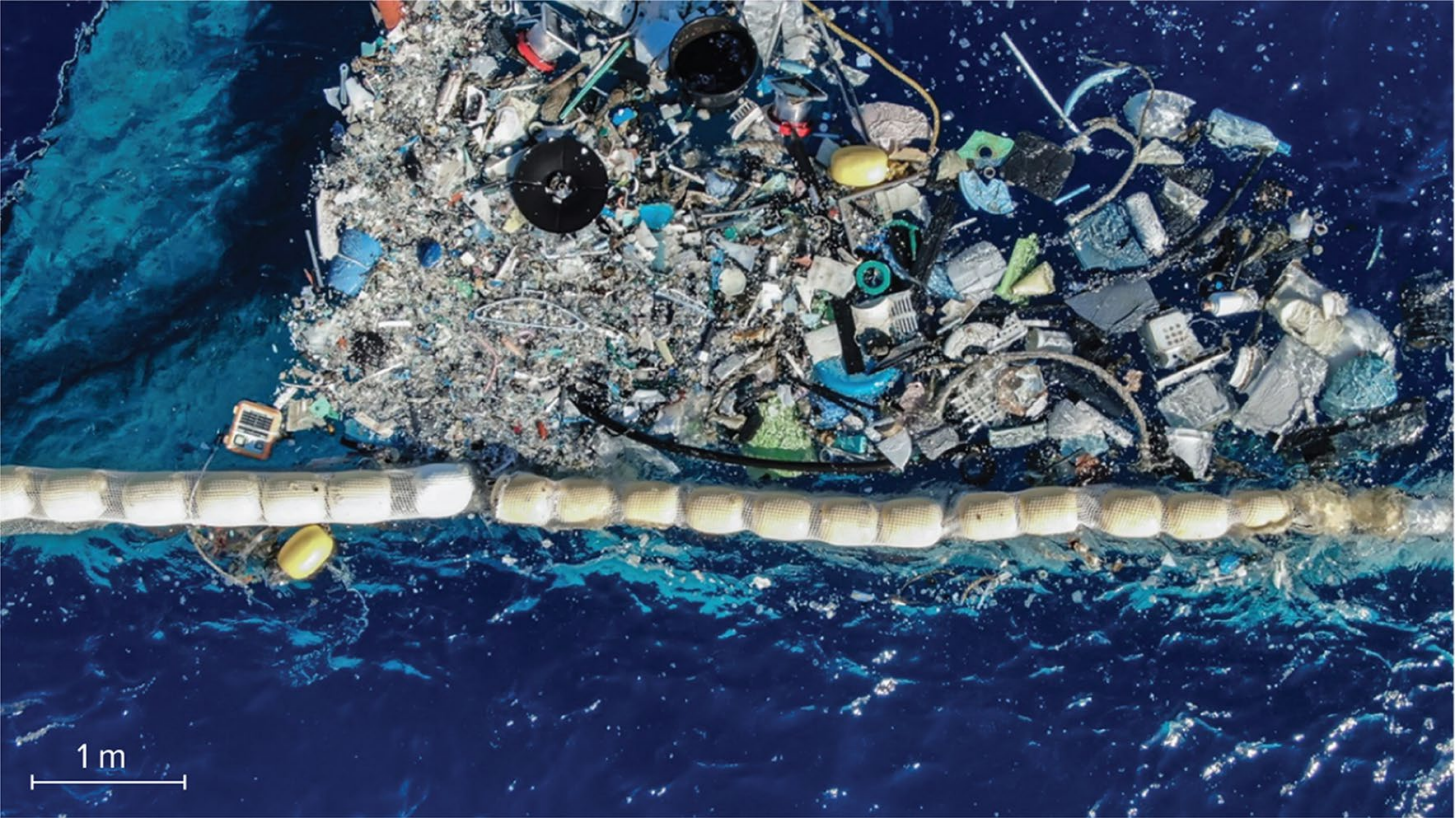
Offshore tests for the recovery of floating plastics conducted by The Ocean Cleanup in the North Pacific Garbage Patch in 2019. Photo credit: Fedde Poppenk.

In this study, we analysed a total of 547 kg of hard plastic debris items retrieved from the NPGP during a campaign of technology tests for the recovery of floating plastic debris offshore by The Ocean Cleanup (Fig. 1), a Dutch non-profit organization developing and scaling technologies to remove floating plastics from the ocean. Particularly, we focussed on countries of origin identified from evidence on hard plastic debris (> 5 cm). Assuming that those items originated from those countries, we tested different land-based and fishing activities source scenarios with a global Lagrangian dispersal model for the transport of floating marine debris to identify the pathways leading to the accumulation of debris in the NPGP. This allowed us to determine the possible locations of sources emitting plastic pollution in this part of the global ocean. Our analyses show that adding to the large fraction of accumulated fishing nets floating at the surface in the region, the majority of floating hard plastics (> 5 cm) accumulated in the NPGP are also likely coming from industrialised fishing nations, thus providing evidence that fishing activities are mostly responsible for the accumulation of floating plastics in the North Pacific subtropical gyre. This information is important as it can inform future mitigation policies, as well as foster participation from the fishing industry and greater cooperation between those nations to monitor and limit the generation of ALDFG in the ocean.

## Methods

### Classification of hard plastics

Offshore plastic debris was collected from the NPGP during The Ocean Cleanup’s System 001/B operations for a series of tests of a recovery system occurring between the 27th of June 2019 and the 7th of November 2019, at latitudes between 33.0° N and 35.1° N and longitudes between 143.0° W and 145.6° W (https://theoceancleanup.com/milestones/system001/). Directly upon retrieval, the collected debris was divided into two fractions: (1) hard plastics (i.e., rigid objects) and (2) nets & ropes, and subsequently stored in individual large bags inside two separate onboard containers. Back onshore, the containers were shipped to the Netherlands and the debris was dried at ambient air temperature. While the oceanographic expedition retrieved a majority of nets and ropes, we focused our analysis on the hard plastics fraction that can carry evidence of their age and geographical origins.

The hard plastics fraction, consisting of 22 large bags, was analysed, following an adapted version of the Litter-ID protocol^40^. The content of each bag was carefully emptied onto a clean surface and the debris items, with the largest dimension > 5 cm, were first individually sorted into 112 predefined categories across nine different material types (Supplementary Table S1) following the OSPAR Beach Litter Monitoring Guideline^41^ allowing for future comparison with beach cleanup data. Then, subcategories within the predefined categories were also added to account for debris items frequently observed in the NPGP such as eel traps, for example. Accordingly, both intact items and fragments with an identifiable item category, such as pieces of a crate were allocated to the corresponding item category or subcategory. Thus, the item counts per item category do not necessarily reflect the number of complete objects in that category, but instead, represent the total counts of intact objects and object fragments combined. Items within the category of unidentifiable fragments were further categorized into seven size classes based on the size classification provided by Lebreton et al.^3^ (< 0.5 mm, 0.5–1.5 mm, 1.5–5 mm, 0.5–1.5 cm, 1.5–5 cm, 5–50 cm and > 50 cm) using a stainless-steel sieve tower. A detailed list of all item categories considered in this study is presented in Supplementary Table S2. Exemplary photos of all plastic item types encountered in the hard plastics fraction are shown in Supplementary Table S3. The items within each OSPAR category were inspected individually for evidence of country of origin (language, company name, brand, logo, other text such as an administrative name, etc.) and production date, and subsequently photographed, counted, and weighed. An origin was attributed from evidence of language only if the language was spoken in one single country (e.g. Japanese). English or Spanish language was excluded as a possible source origin by itself due to its universal use. Native speakers assisted us with the identification of Asian languages. Sometimes a kanji was identified that could have been used in both Chinese and Japanese. In that case, no origin was attributed to the object. An origin could also be determined from a logo or company name unless the brand was established internationally (i.e. with offices in more than one country). We investigated brands that were unknown to us on Google search engine and subsequently visited the brand’s website to identify in which country the company was established. The country of origin may provide clues as to the source and/or location where the item entered the marine environment. For fragments < 5 cm, only the weight was taken as these fragments were too numerous and too small to be counted individually or to be investigated for evidence of origin and age. In addition, fragments < 5 cm contained small pieces of gooseneck barnacle shells and therefore did represent not only plastic items but also biogenic debris. While the weights for these fragments are reported in the Supplementary Information (Supplementary Table S2), they are excluded in the analysis performed here to allow for a consistent methodology and thus comparability of all item categories.

### Lagrangian dispersal modelling and source scenario distribution

To understand how and where floating plastics found in the NPGP enter the ocean, we implemented a series of global Lagrangian dispersal simulations of floating marine litter transport that we compared with our composition analysis. Lagrangian dispersal models are useful tools to study the connectivity at the surface of the ocean and transport of floating marine debris^42^. The model framework is documented in Lebreton et al.^43^. In short, floating plastics are represented by Lagrangian particles advected by data on sea surface currents and released in time from representative source distributions at a global scale (Supplementary Table S4). In this study, we simulated continuous inputs from 2013 to 2019 using ocean circulation data from the HYCOM/NCODA 1/12-degree global reanalysis^44–46^, and we extracted the modelled particles present in the NPGP region, simplified as the area of longitudes between 160.0° W and 130.0° W and latitudes between 20.0° N and 50.0° N, and for November 2019, corresponding to the date of completion of our oceanographic mission. No additional windage effect was applied on the trajectory of modelled particles as debris accumulating in the subtropical gyre is better represented with low sea surface wind forcing^3,47^. Our search area encompassed a larger area than the actual accumulation zone to allow for geographical variability in the central position of the NPGP, however 97% of particles used to derive our results were contained in the inner accumulation area. The 7-year simulation coverage was motivated by the availability of mapped fishing effort derived from AIS signals recorded by satellites^48^ and distributed by the Global Fishing Watch (https://globalfishingwatch.org). Particularly the dataset differentiates between fishing gear and vessel flags. In this study, we represented inputs for the nine main categories of fishing techniques reported globally (drifting longlines, seiners, trawlers, pole and line, trollers, fixed gear, dredge fishing, squid jigger and, unknown/unidentified fishing). Each year a particle was released from every 0.1 × 0.1-degree cell of the global ocean where a gear and country-specific fishing effort exceeding 15 min was recorded. The simulation represented the dispersal of 9,994,224 particles corresponding to 250,691,680 h of fishing effort between 2013 and 2019 (Supplementary Table S5). Since no estimate for plastic mass input per unit of fishing effort was available, particles were assigned the total fishing effort in number of hours recorded by locations, assuming that the longer the effort the higher the likelihood of ALDFG emissions. To compare our fishing source scenario with other global estimates of plastic inputs into the ocean, we implemented another source distribution into our model based on macroplastic inputs from rivers^8^. To acknowledge the large uncertainties associated with the estimate of plastic emissions using country scale municipal solid waste data^49^, two additional scenarios were created by (1) taking river inputs from Meijer et al.^8^ and (2) by adjusting for values of mismanaged plastic waste (MPW) generation per countries from two other global studies from Borrelle et al.^7^ and Chen et al.^50^ predicting plastic waste discarded on land. In the case of river inputs, one Lagrangian particle was released for every tonne of plastic emissions. Annual emissions between 2013 and 2019 were further scaled with data on annual plastic production^51^.

Modelled Lagrangian particles carried information on country of origin derived from the location of inputs for the different river scenarios and from the vessel flag for the fishing source scenario, allowing us to simulate the respective contribution per scenario and per country of any region accumulating floating plastics globally. By looking at model particles accumulated in the North Pacific subtropical gyre at the end of 2019, we were able to formulate the contribution into the NPGP per country for the different source scenarios. For both river and fishing sources scenarios, we identified the country of origin or vessel flag of particles present in the NPGP region after seven years of simulation and derived the contributions of each country. To score the different scenarios, we computed the coefficient of determination R^2^ between observed and modelled contributions of the main fishing nations identified in the region (Japan, China, Korea, USA, Taiwan and Russia, Supplementary Fig. S1) as well as another category for other countries classified as “others”.

### Particle trajectory analysis and beaching of debris

We investigated the role of beaching and analysed individual trajectories of modelled Lagrangian particles for both rivers and fishing source scenarios. For every trajectory, we counted the total time spent by particles near coastlines. A particle was considered next to a coastline when it was located at a distance smaller than the ocean circulation model cell size (1/12 degree, less than 10 km). We then classified the particles extracted from the NPGP region for each scenario by the total amount of time spent next to a coastal cell. The dynamics of floating plastics in nearshore areas are largely unconstrained at a global scale due to the complexity of processes (tides, waves, wind, freshwater plumes, interaction with biota…) and variations between coastlines (nearshore slope, coastal morphology, substrate, coastal development…)^23^. As such, current models typically simplify the beaching process by assuming that the longer floating plastics spend in coastal regions the more likely they are removed from the sea surface. Thus, to quantify mass-loss rates (*f*_*beach*_) from beaching of plastic debris, we included a sink term from beaching probability (*P*_*beach*_) as defined in Kaandorp et al.^12^:

$${f}_{beach}=1-{P}_{beach}={e}^{-{t}_{coast}/{\tau }_{beach}}$$

With *t*_*coast*_ representing the total time a particle spends next to a coastal cell and *τ*_*beach*_ equalling to the characteristic beaching time scale. In the simulation, model particles are not allowed to beach but the longer the time they spend next to a coastline, the lesser their contribution to offshore accumulation. The beaching timescale can be interpreted as the time for which particles are permanently stored on the coastline due to burial, sinking or entrapment, and not further released into the ocean. Available estimates for *τ*_*beach*_ vary between 24 days as determined by inverse dispersal modelling for the Mediterranean Sea^12^, and 2 days (i.e., at least one full tidal cycle) as previously suggested for the global scale^9^.

## Results

### Debris classification

**Figure 2:**
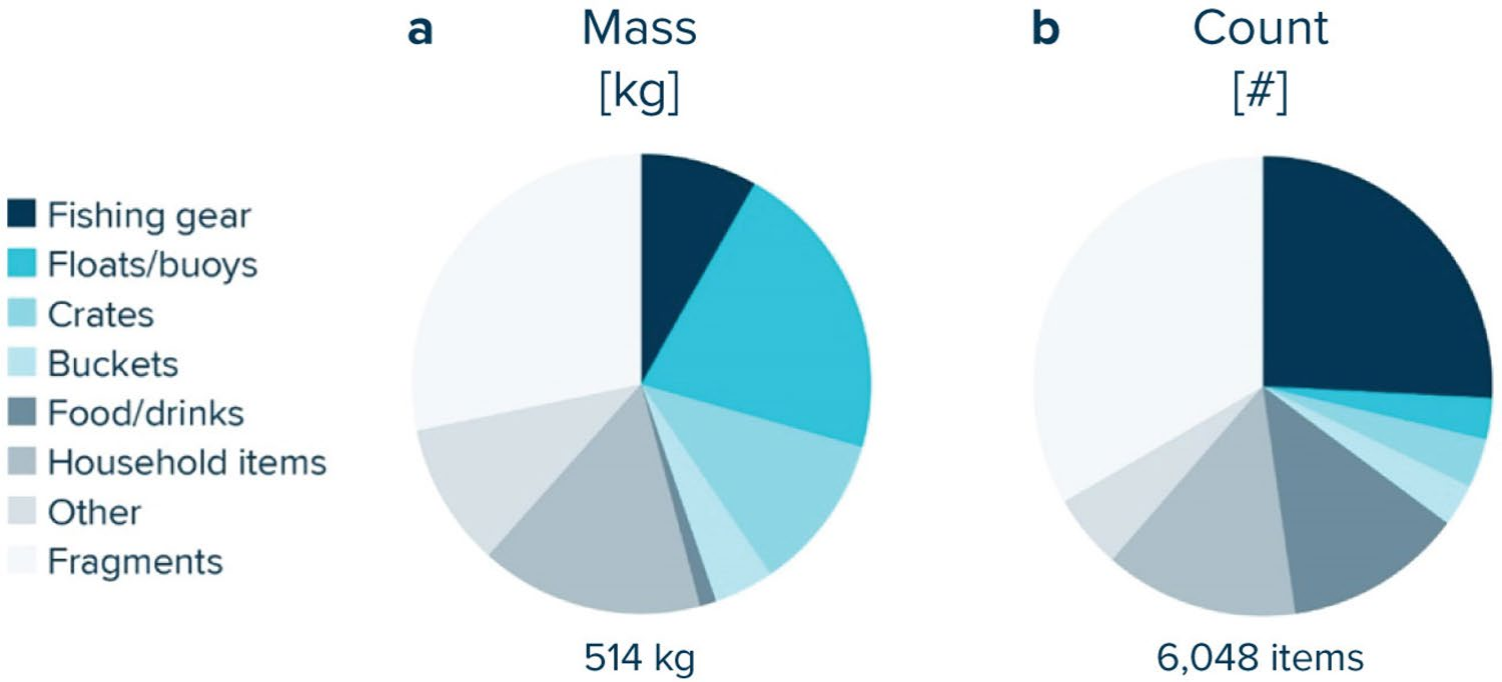
Composition of hard plastic debris harvested from the North Pacific Garbage Patch in 2019. Relative (**a**) mass and (**b**) numerical distribution of hard plastic items > 5 cm only (e.g., excluding nets and ropes).

In total, 6,093 debris items > 5 cm made of different materials and collected from the NPGP were analysed individually amounting to a total (dry) weight of 573 kg (Supplementary Table S1). With 6,048 items > 5 cm documented, plastic accounted for > 99% of the rigid items by count and represented 90% of the total debris mass (514 kg). Most common plastic objects were unidentifiable fragments (33% by count and 28% by mass, Table 1, Fig. 2). Fishing and aquaculture gear such as fish boxes, oyster spacers and eel traps, was the second most common category accounting for 26% of the number of hard plastic objects collected and for 8% of the mass. Plastic floats and buoys contributed to 3% of the number of plastic objects but represented 21% of the total mass. Plastic items associated with food and drinks represented 13% of the total plastic items and were mostly composed of bottle caps and lids. Hence due to their small weight represented only 1% of the total mass. Finally, household items accounted for 14% and 16% of the number and mass of plastic objects, respectively. For this category, most weight was carried by containers, drums, jerry cans and baskets. Some categories of debris like fishing gear or buoys can easily be attributed to fishing activities, but the sources of other categories such as crates, buckets or food packaging can be more difficult to attribute as they could also be coming from land-based sources or other maritime activities.

**Table 1:** Composition of hard plastics > 5 cm collected from the North Pacific Garbage Patch in 2019 and regrouped into plastic item categories.

Material	(#)	(kg)	% (#)	% (kg)
**Aquaculture gear**	**781**	**4.38**	**12.9**	**0.8**
Oyster nets, bags, spacers (28)	779	4.36	99.7	99.5
Oyster trays (29)	2	0.02	0.3	0.5
**Fishing gear (excluding nets and ropes category)**	**781**	**37.89**	**12.9**	**7.4**
Crab/lobster pots (26)	37	0.33	4.7	0.9
Lobster and fish tags (114)	1	0.00	0.1	0.0
Fish boxes (34)	430	30.54	55.1	80.6
Light sticks (36)	3	0.05	0.4	0.1
Eel traps (48L)	310	6.97	39.6	18.4
**Floats/buoys (37)**	**173**	**108.80**	**2.9**	**21.2**
**Crates (13)**	**208**	**56.81**	**3.4**	**11.1**
**Buckets (38)**	**183**	**21.93**	**3.0**	**4.3**
**Food/drinks**	**760**	**5.88**	**12.6**	**1.1**
Drinks (bottles, containers, and drums) (4)	9	0.20	1.2	3.4
Food containers incl. fast food containers (6)	24	2.08	3.2	35.3
Caps/lids (15)	726	3.60	95.5	61.3
Crisp/sweet packets and lolly sticks (19)	1	0.00	0.1	0.0
**Household items**	**826**	**80.85**	**13.7**	**15.7**
Plastic bag ends (112)	2	0.02	0.2	0.0
Cleaner (bottles, containers, and drums) (5)	45	2.04	5.4	2.5
Cosmetics (e.g., sun lotion, shampoo, shower gel) (7)	21	0.86	2.5	1.1
Engine oil containers and drums (8)	34	1.63	4.1	2.0
Jerry cans (10)	63	18.14	7.6	22.4
Injection gun containers (11)	8	0.44	1.0	0.5
Other bottles, containers, and drums (12)	302	29.52	36.6	36.5
Car parts (14)	10	1.17	1.2	1.4
Cigarette lighters (16)	2	0.02	0.2	0.0
Pens (17)	21	0.07	2.5	0.1
Combs/hairbrushes (18)	7	0.03	0.8	0.0
Toys & party poppers (20)	44	1.18	5.3	1.5
Cutlery/trays/straws (22)	7	0.09	0.8	0.1
Hard hats (42)	2	0.16	0.2	0.2
Shotgun cartridges (43)	5	0.02	0.6	0.0
Shoes/sandals (44)	1	0.08	0.1	0.1
Sanitary waste (98, 101–102)	65	0.34	7.9	0.4
Medical waste (104–105)	3	0.01	0.4	0.0
Pipes/tubes (48B)	62	4.50	7.5	5.6
Electrical wire (48H)	62	0.10	7.5	0.1
Plastic cleaning brush (48I)	5	0.23	0.6	0.3
Baskets (48K)	55	20.21	6.7	25.0
**Other**	**319**	**52.26**	**5.3**	**10.2**
Conveyor belt items vessel (48D)	13	0.26	4.1	0.5
Detonation chord (48G)	3	0.00	0.9	0.0
Melted/burned (48J)	182	7.39	57.1	14.1
Other plastic items (48M)	121	44.61	37.9	85.4
**Fragments**	**2017**	**145.12**	**33.3**	**28.2**
Unidentifiable fragments (5–50 cm) (117F)	1964	40.71	97.4	28.1
Unidentifiable fragments (> 50 cm) (117G)	35	104.33	1.7	71.9
Styrofoam small (< 5 cm) (48E)	10	0.00	0.5	0.0
Styrofoam large (> 5 cm) (48F)	8	0.07	0.4	0.1
**Total**	**6048**	**513.91**	**100.0**	**100.0**

Numbers in (brackets) represent OSPAR category ID^41^.

**Figure 3:**
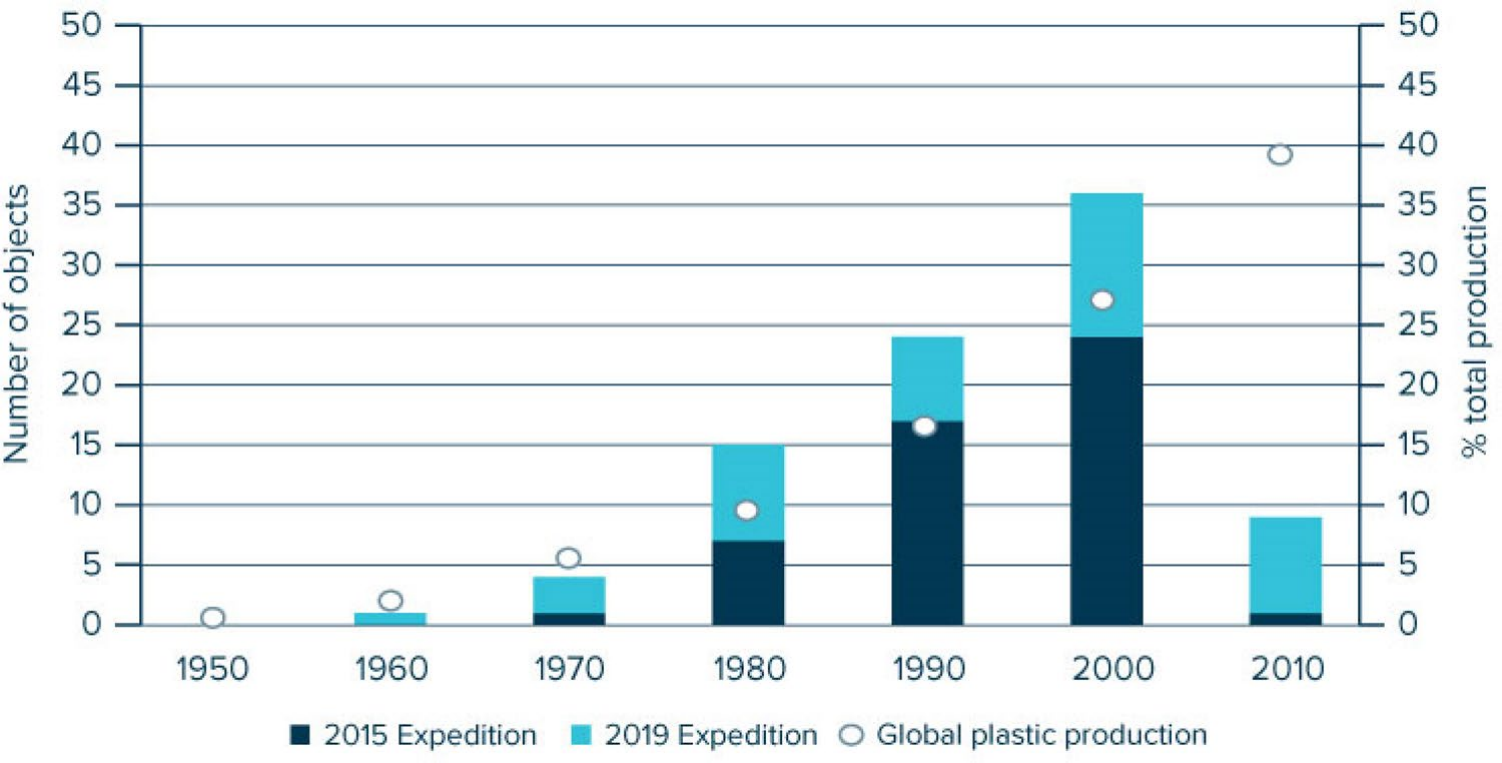
Distribution of production date labels identified on plastic objects collected from the North Pacific Garbage Patch in 2015^3^ (n = 50) and in 2019 (this study, n = 39). See Supplementary Table S8 for joint values with identified countries of origin identified for this study. Dots represent relative distribution of global plastic production per decade^51^. Note that global production for the years 2016–2019 was estimated by extrapolating the exponential production increase as observed during the years 1980–2015 (see Supplementary Fig. S2, Supplementary Table S11).

A total of 201 plastic objects had recognizable language writings on them. The most common languages identified on these plastic objects were Chinese (34%), Japanese (33%), English (17%) and Korean (10%) (Supplementary Table S6). Furthermore, a total of 232 plastic objects had an identifiable origin based on evidence such as language, text, company name or brand. The origin of 19 objects with an identified language could not be determined (12 objects with English markings and 2 objects with Spanish markings but with no further evidence, and 5 objects with a kanji that could have been Japanese or Chinese). The origin of 42 objects with no language identified was determined mostly by identifying a local brand name or a logo. Inversely, 101 objects with or without language had an identified brand but no origins were attributed as the brands were established internationally. The top five identified origins were Japan (34%), China (32%), Korea (10%), USA (7%) and Taiwan (6%) (Supplementary Table S7). The identification of these countries of origin is consistent with previous findings from an expedition in the NPGP in 2015^3^. The identification of production dates also replicated previous results, with nearly half (49%) of identified production dates found on objects from the twentieth century while the oldest identified item was a buoy dating from 1966 (Fig. 3). The complete list of floating plastics objects (n = 354) retrieved during the 2019 expedition and with identified evidence of brand, language, origin, or age, is publicly available on FigShare^52^.

### Comparison with dispersal modelling scenarios

The correlations between our modelled and observed NPGP hard plastic origins were generally higher with the fishing source scenario than with any land-based scenario (Table 2), further suggesting that a large fraction of floating hard plastics (> 5 cm) in the NPGP may also be coming from fishing activities and not directly from land. China, Japan, South Korea, USA and Taiwan, the five countries mostly represented in our field observations are all active fishing nations in the region as they cumulated 87% of the simulated fishing effort contributing to modelled emissions into the NPGP. The remaining identified fishing effort was mostly coming from Russian vessels (13%). The contribution of these countries was much smaller in the land-based emission scenarios. Particularly other countries at the rim of the North Pacific Ocean such as the Philippines, which were not well represented in our observations at sea, were predicted as major contributors to the NPGP in our land-based emission models (Supplementary Fig. S3). The modelled contribution of Japan was substantially less than in our observations, both for the fishing source scenario as well as for most river scenarios. We explained this by the presence of Japanese debris originating from the 2011 Tohoku earthquake and tsunami which released large amounts of debris into the ocean at once and of which a fraction is still floating in the North Pacific Ocean^47^. As such, we also tested our modelled scenarios against field observations after removing the contribution of Japan as an origin, which led to considerably better results for the fishing source scenario (R^2^ = 0.71) but not for any land-based scenario (Table 2).

**Table 2:** Comparison between identified origins of hard plastic items > 5 cm collected from the NPGP in 2015^3^ and in 2019 (this study) against modelled contribution of countries for different river^7,8,50^ and fishing effort^48^ scenarios.

Observations	Japan	China	Korea	USA	Taiwan	Russia	Others		
# plastic items 2015	124	114	64	16	5	1	25		
# plastic items 2019	78	75	23	15	13	1	27		
Total	202	189	87	31	18	2	52		
%	35	33	15	5	3	0	9		

								R^2^	R^2^*
**Modelled river sources contributing to the NPGP (2013–2019)**									
Meijer et al.^8^	3%	10%	0%	1%	1%	0%	86%	0.01	0.00
Borrelle et al.^7^	54%	4%	1%	2%	0%	5%	34%	0.27	0.00
Chen et al.^50^	26%	2%	4%	13%	12%	4%	41%	0.00	0.07
**Modelled fishing effort contributing to the NPGP (2013–2019)**									
Trawlers	1%	73%	1%	7%	1%	16%	1%	0.22	0.68
Fixed gear	2%	56%	7%	12%	3%	19%	1%	0.16	0.61
Drifting longlines	26%	10%	14%	20%	25%	0%	6%	0.08	0.01
Others/unidentified	5%	72%	5%	5%	3%	7%	4%	0.32	0.80
All fishing	5%	61%	5%	9%	5%	13%	2%	0.26	0.71

The contributions are weighted with mass inputs in tonnes for the river scenarios and in number of fishing hours for the fishing effort scenario. See Supplementary Table S10 for absolute values. Modelled contribution of countries (Supplementary Fig. S3) is rated by scenarios (Supplementary Fig. S1) and coefficient of determination R^2^ and R^2^* are reported for regressions between model and observations, made respectively with and without the contribution of Japan.

**Figure 4:**
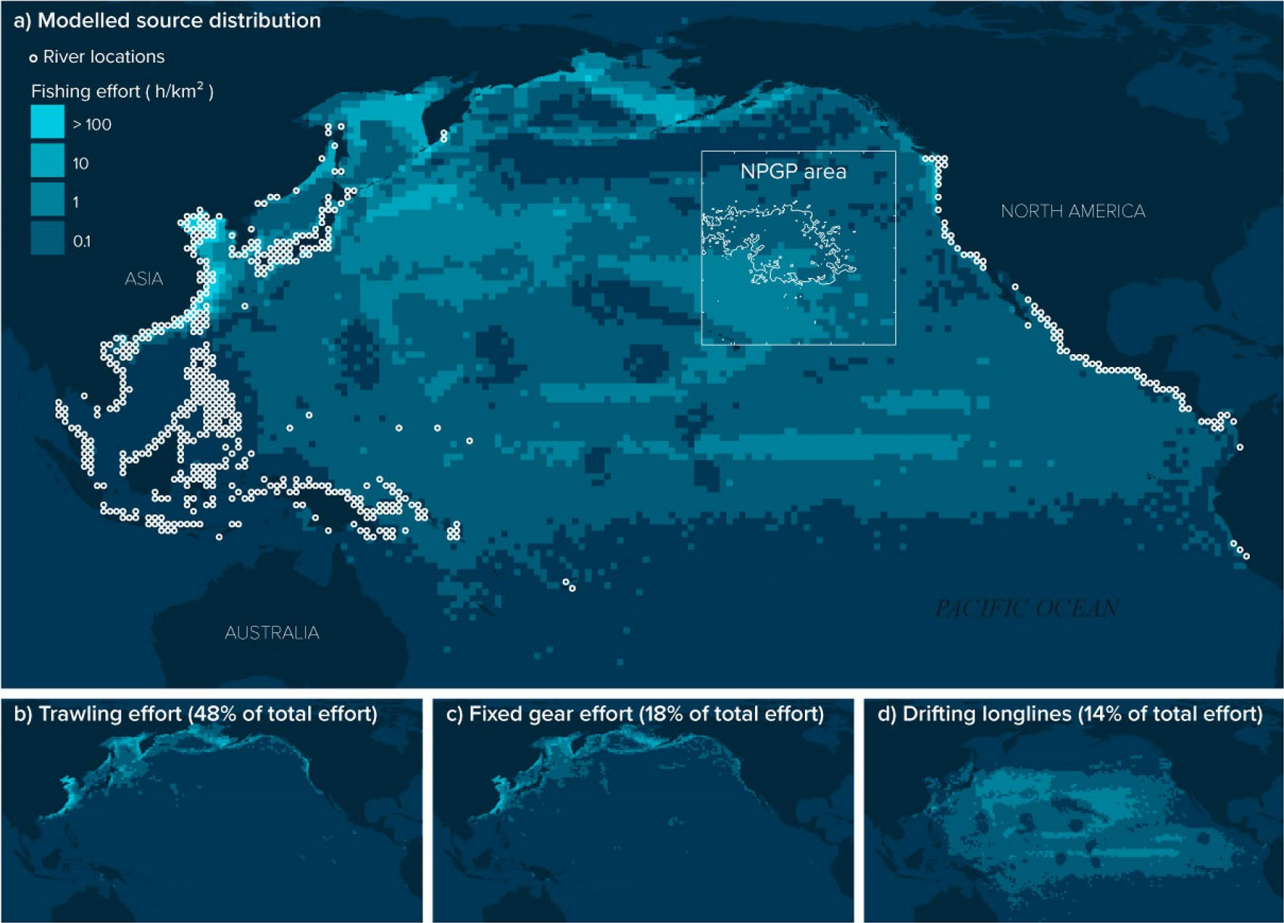
Land-based and maritime distribution of modelled sources of floating plastic debris found in the NPGP area. (**a**) Lagrangian particles detected in the NPGP area (white square, 97% of particles were detected inside the white contour line) were initially released either from river mouth locations^8^ (white circles) or from observed fishing grounds^48^ quantified by the level of fishing effort in h/km^2^ from vessels equipped with AIS. Fishing effort was differentiated by type of gear. Three fishing techniques, out of nine simulated, represented most of the identified effort connected to the NPGP: trawling (**b**), fixed gear (including set nets, set longlines, traps and pots) (**c**) and drifting longlines (**d**) with respectively 48%, 18% and 14% of the total simulated effort carried by particles found in the NPGP area in 2019. These maps were generated using QGIS version 3.8.3 (www.qgis.org).

The fishing source scenario gave us insights on countries of origin but also on fishing techniques that could contribute to ALDFG found in the region. The simulated global fishing effort differentiated between nine different fishing gear categories, and we focused our analysis on the three most represented categories (Supplementary Table S9). Trawlers cumulated 48% of fishing effort that contributed to model particles found in the NPGP while fixed gear and drifting longlines totalled 18% and 14%, respectively (Supplementary Table S9). For 16% of modelled fishing effort contributing to model particle emissions, the technique was unidentified and could have also been representative of one of these three gear categories. As such, trawlers, fixed gear, and drifting longlines accounted for more than 95% of identified fishing effort that could have accounted for emissions of floating plastic debris from fisheries into the NPGP. Trawling and fixed gear effort contributing to the NPGP generally occurred near the Asian and North American continental shelves while drifting longlines effort was distributed throughout the oceanic zone of the whole North Pacific Ocean (Fig. 4).

#### Model trajectory analysis and probability of reaching the NPGP

**Figure 5:**
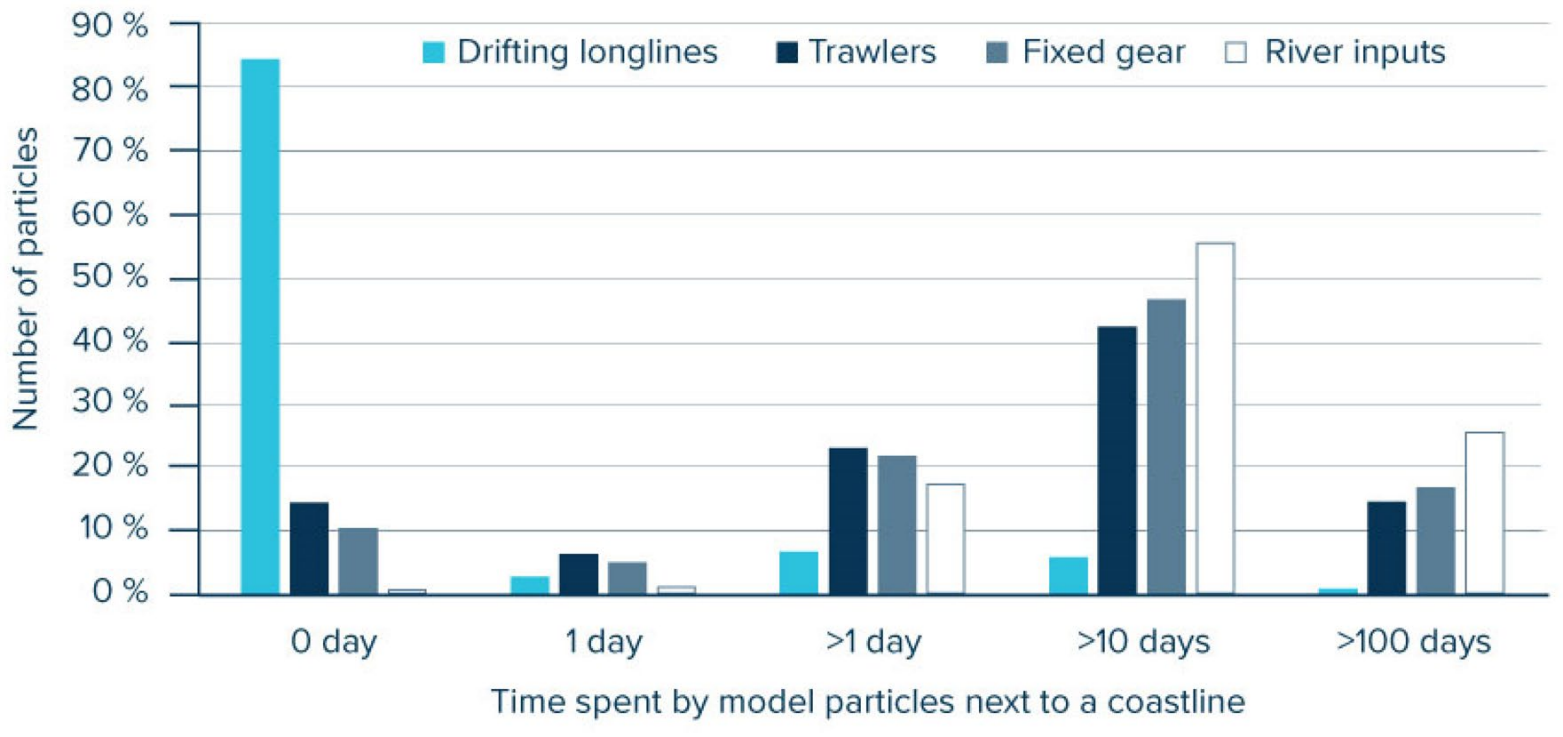
Distribution of modelled Lagrangian particles found in the North Pacific Garbage Patch area by total amount of time spent near a coastline for three different fishing gear scenarios and for the baseline river scenario^8^. With more time spent near a coastline, the beaching probability for floating plastics is increasing.

To understand why land-based input scenarios poorly represented identified origins of floating plastic debris collected in the NPGP, we investigated the role of beaching on trajectories of modelled Lagrangian particles. Simulated debris originating from rivers generally spent more time near shorelines with 81% of modelled particles having spent more than 10 days and 26% more than 100 days in proximity to a coastline prior to reaching the NPGP (Fig. 5). Only 2% of modelled particles from river sources spent one or less than a day next to a coastline. In comparison, 21% and 15% of particles released from trawling and fixed gear effort respectively had spent one or less than a day next to a coastline. As drifting longlines effort generally occurred offshore, modelled particles for this gear spent very little time next to a coastline with more than 85% of particles not encountering land during the simulation. By computing the decreasing probability of sea surface dispersion with time spent next to a coastline, we can evaluate the impact of beaching and compare the fate of the same mass of plastic emitted from rivers or from fishing sources at a regional scale. Using a characteristic beaching time scale (*τ*_*beach*_) of 24 days for marine debris as it was previously estimated for the Mediterranean Sea^12^, we estimated that in the North Pacific, positively buoyant plastics emitted from fishing activities were nearly twice more likely (i.e., 187%) to reach the subtropical waters than plastics originating from rivers. Under this modelled beaching scenario, every kilogram of floating hard plastic released in the North Pacific would result in 0.58 kg reaching the subtropical gyre when released from fishing activities and in 0.32 kg when released from rivers. With a characteristic beaching time of two days, a period of time longer than a full tidal cycle as previously proposed at a global scale^9^, the probability of a debris item reaching the NPGP was more than ten times (i.e., 1159%) higher for fishery sources than for river sources. Under this modelled beaching scenario, every kilogram emitted from rivers resulted in 0.03 kg reaching the subtropical gyre while every kilogram emitted from fisheries resulted in 0.33 kg of inputs to NPGP.

## Discussion

In this study, we provide new insights into the composition, sources and origins of floating plastic debris accumulating in the NPGP by combining waste composition analysis, global fishing effort observations and Lagrangian dispersal modelling. Our results replicated those of a previous analysis of 223 kg of hard plastics (> 5 cm) retrieved in the same area in 2015 by The Ocean Cleanup^3^. A large fraction of the plastic mass accumulating in these offshore waters is carried by a few objects made in the vast majority of floating nets and ropes, several meters in size. Smaller hard plastic objects also represent a substantial amount of accumulated floating plastic mass^3^. These hard plastics carry valuable information on their use and origin, allowing a better understanding of the origin and source of emissions as well as the transport and fate of persistent floating plastic marine debris. Our new results indicate that a significant fraction of these hard plastics may also be coming from fishing vessels. Adding to the mass of floating nets and ropes, this suggests that between 75 and 86% of the floating plastic mass (> 5 cm) in the NPGP could be considered ALDFG. With our results, we show that five countries mostly contributed to the formation of the NPGP, with most identified emissions originating from Japan, China, South Korea, the USA and Taiwan. These five countries were not recognised as major contributors to land-based emissions of plastics into the ocean but instead, they were identified as major fishing nations in the North Pacific Ocean. This conclusion comes from the analysis of hard plastic debris found in the NPGP but it is likely also applicable to nets and ropes for which the origin is harder to determine. Our findings further highlight that fisheries play an important role in the solutions to the ocean plastic pollution problem.

Here, we investigated the discrepancy between estimated land-based emissions of plastics and observed accumulation in an offshore area dominated by ALDFG. We explained this apparent discrepancy by a higher likelihood of floating plastic debris emitted from fisheries reaching the subtropical gyres compared to floating plastic debris originating from land-based sources. Floating plastics emitted from the coast depict a much greater chance to rapidly return to land with most litter stranding within a short distance of the river mouth^13,53^. Furthermore, floating plastic items escaping rivers into the ocean mostly differ from the type of hard plastic debris found in subtropical gyres^29^, i.e., thick positively buoyant plastics made of polyethylene and polypropylene. These plastics represented less than 15% of observed plastics flowing at the surface of European and Asian rivers^54^ suggesting that the rest rapidly fragments, beach onto coastlines, and/or sinks to the bottom of the coastal ocean.

The compositional differences of plastic pollution between coastal and offshore waters therefore suggest that plastics originating from land are predominantly trapped in nearshore areas and may be eventually released to the open ocean as small, degraded plastic fragments^29^. Plastic fragments (< 5 cm) represented 21% of the predicted mass of accumulated plastic in the NPGP^3^. However, it is difficult to know which proportion of these unidentifiable fragments and smaller particles is coming from the fragmentation of larger plastics already accumulated in the area, and which fraction was transported from land sources already in that form.

Furthermore, with the investigation of several river source scenarios for macroplastics, we also highlight the variability between global estimates of inputs from land to the ocean. Inconsistencies related to estimating plastic waste generation from national statistics on municipal solid waste often result in these discrepancies^49^. As such, large uncertainties remain with quantifying inputs of plastics from land into the ocean. It is also unclear how large the contribution of extreme events such as flooding^55^ or tsunamis^47^ is to oceanic plastic pollution. Finally, other marine sources such as aquaculture^56^ and shipping activity^21^ that were not considered in our study can also contribute to ocean plastic pollution. For instance, our analysis revealed a large number of plastic objects (n = 781) used in oyster farming. However, these objects, mostly oyster spacers (> 99%), were relatively light and therefore contributed only a small fraction (< 1%) of the total mass of floating plastic debris in the NPGP.

The identification of debris origin was based on a mix of evidence from the identification of language, brand, logo and other clues such as a simple address. However, some brands are established internationally, or some languages are used universally so some uncertainty remains. As such we conservatively removed from our analysis, every object for which the origin was ambiguous. Yet the results from two separate expeditions in 2015 and 2019 showed the same trend of possible origins, increasing our level of confidence.

Japan was the most identified country of origin for floating plastics collected from the NPGP in both 2015 (36%^3^) and in 2019 (34%, this study). We attribute this observation to inputs from fisheries with Japan being a major fishing nation but also to the anthropogenic debris released by the 2011 Tohoku tsunami. We noted eight plastic objects originating from Japan on which a production date was visible (Supplementary Table S11). All eight objects were produced prior to 2011, with the newest object being from 2007 and the oldest from 1975. It is difficult to differentiate debris originating from continuous inputs versus debris released during one single extreme event.

Our results highlight the complexity of sources and transport of floating plastics in the ocean where different types of positively buoyant plastic objects will have a different fate as a function of size and composition but, importantly, also of release location. The recovery of plastic debris in subtropical gyres is a challenging endeavour. While these efforts help in reducing the mass of plastic accumulated at the surface of the ocean, they also enable the analysis of debris composition and origin allowing to identify the sources of pollution which is essential to design mitigation measures aiming at reducing future inputs. In this study, we provide an explanation for the dominance of ALDFG in plastic material accumulated at the surface in the North Pacific subtropical gyre, which is a remote and offshore part of the Pacific Ocean. A greater transparency from the fishing industry and strengthened cooperation between countries to regulate and monitor the generation of ALDFG would help reduce emissions from the ‘other tap’ of ocean plastics.

## Supplementary Information

Supplementary Information.
