# Supplementary material for: Industrialised fishing nations largely contribute to floating plastic pollution in the North Pacific subtropical gyre
Source: Sci Rep. 2022 Sep 1;12:12666. doi: 10.1038/s41598-022-16529-0 (PMC9436981; doi:10.1038/s41598-022-16529-0)
Supplement: Supplementary file 1 — Supplementary Information. [file 41598_2022_16529_MOESM1_ESM.docx]

**Supplementary Material for ‘Industrialised fishing nations largely contribute to floating plastic pollution in the North Pacific subtropical gyre’**

**Laurent Lebreton^1,2,*^, Sarah-Jeanne Royer^1^, Axel Peytavin^1^, Wouter Jan Strietman^3^, Ingeborg Smeding-Zuurendonk^3^, Matthias Egger^1,4^**

^1^The Ocean Cleanup, Rotterdam, The Netherlands

^2^The Modelling House, Raglan, New Zealand

^3^Wageningen University & Research, Wageningen, The Netherlands

^4^Egger Research and Consulting, St Gallen, Switzerland

*Corresponding author: laurent.lebreton@theoceancleanup.com

**Supplementary Figures**


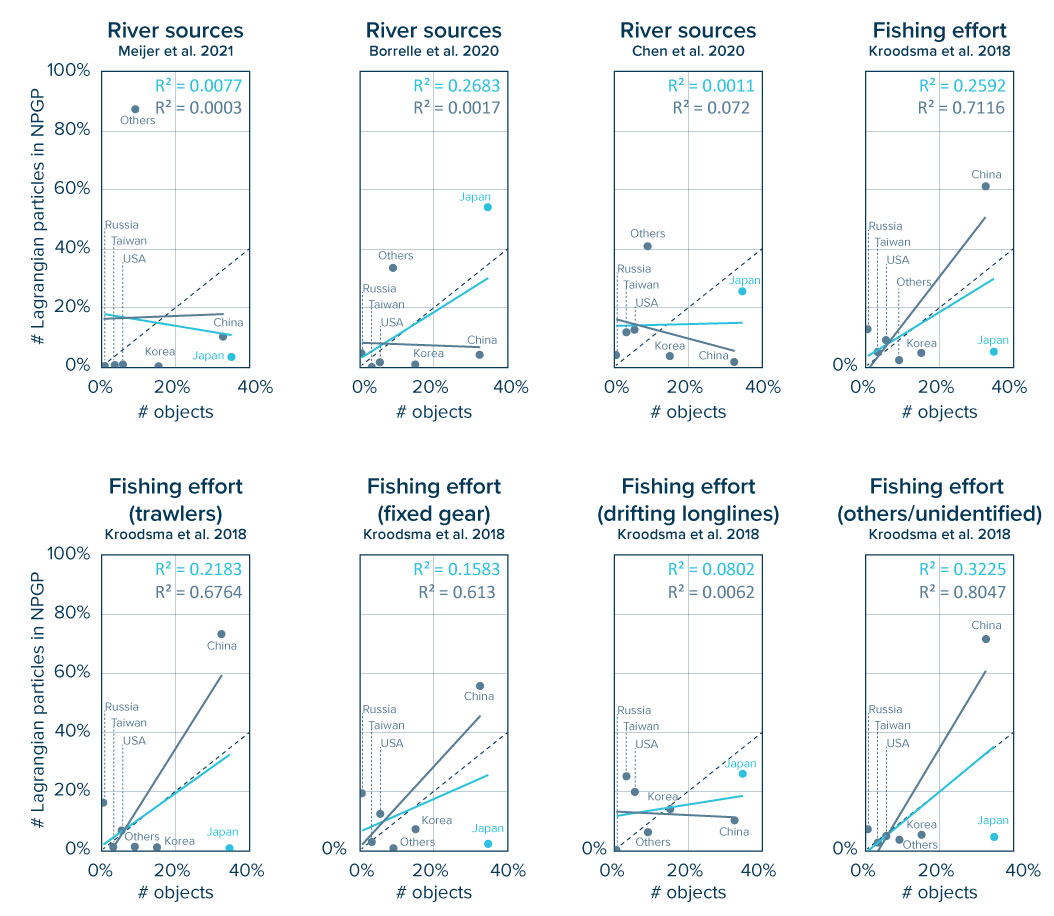


**Figure S1 |** Regression analysis between origins by number of objects retrieved from the NPGP and the contribution by countries for different Lagrangian modelling scenarios. The results are provided for no beaching scenario (τ_beach_ = ∞). The relative modelled contribution per country with different beaching scenarios is very similar, and beaching has mostly an effect on the absolute quantities reaching the NPGP. The coefficient of determination is calculated with (light blue) and without (grey) the contribution of Japan to account for the bias introduced by debris originating from the 2011 tsunami.

**
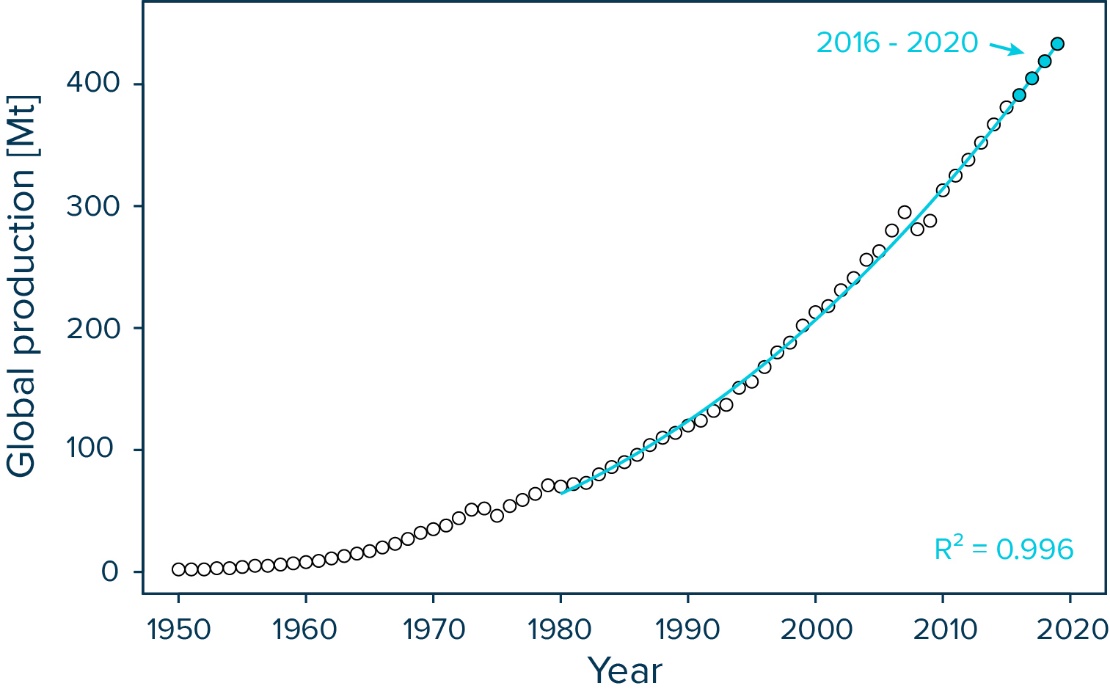
**

**Figure S2 |** Global plastic production since 1950 in million tonnes (Mt). The white dots represent values between 1950 and 2015 as reported in Geyer et al. (2017)^1^. The blue dots represent values for the years 2016-2019 (i.e., 391, 405, 419, and 433 Mt) estimated by extrapolating the exponential trend (blue line; R^2^ = 0.996) between the years 1980 to 2015.


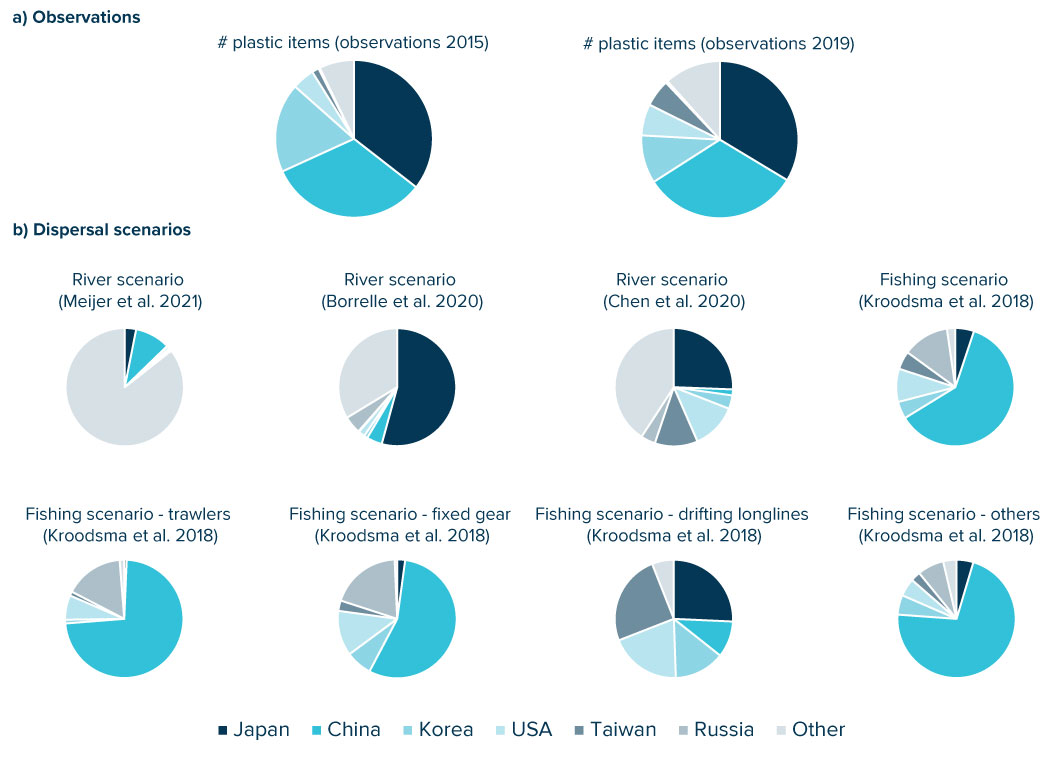


**Figure S3 |** Contribution by countries for (a) origins identified on debris collected in the NPGP and (b) modelled particles in the NPGP for different dispersal scenarios.

**Supplementary Tables**

**Table S1 |** Composition of hard debris > 5 cm (e.g., excluding nets and ropes) collected from the North Pacific Garbage Patch in 2019 regrouped into OSPAR material categories.

| **Material** | **[#]** | **[kg]** | **% [#]** | **% [kg]** |
| --- | --- | --- | --- | --- |
| Plastic / Polystyrene | 6048 | 513.91 | 99.26 | 89.68 |
| Rubber | 4 | 0.08 | 0.07 | 0.01 |
| Cloth | 3 | 0.09 | 0.05 | 0.02 |
| Paper / Cardboard | 5 | 0.00 | 0.08 | 0.00 |
| Wood | 2 | 0.01 | 0.03 | 0.00 |
| Metal | 11 | 53.67 | 0.18 | 9.37 |
| Glass | 14 | 4.70 | 0.23 | 0.82 |
| Ceramics | 1 | 0.54 | 0.02 | 0.09 |
| Other | 5 | 0.04 | 0.08 | 0.01 |
| **Total** | **6093** | **573** | **100.00** | **100.00** |

**Table S2 |** Item counts [#] and associated weights [kg] of hard debris collected from the NPGP during The Ocean Cleanup’s System 001/B operations in 2019. The item categories are based on the OSPAR Beach Litter Monitoring Guideline^2^. Material categories are highlighted in grey.

| **OSPAR ID** | **Items** | **# items** | **Weight [kg]** |
| --- | --- | --- | --- |
|  | **Plastic / Polystyrene** | **6048** | **546.797** |
| 1 | 4/6-pack yokes | 0 | 0.000 |
| 2 | Bags (e.g., shopping) | 0 | 0.000 |
| 3 | Small plastic bags, e.g., freezer bags | 0 | 0.000 |
| 112 | Plastic bag ends | 2 | 0.018 |
| 4 | Drinks (bottles, containers, and drums) | 9 | 0.197 |
| 5 | Cleaner (bottles, containers, and drums) | 45 | 2.043 |
| 6 | Food containers incl. fast food containers | 24 | 2.077 |
| 7 | Cosmetics |  |  |
| 7A | Shampoo bottles | 12 | 0.353 |
| 7B | Sun lotion | 4 | 0.413 |
| 7C | Deodorant | 2 | 0.054 |
| 7D | Shower gel | 0 | 0.000 |
| 7E | Other cosmetics bottles / containers | 3 | 0.045 |
| 8 | Engine oil containers and drums < 50 cm | 34 | 1.632 |
| 9 | Engine oil containers and drums > 50 cm | 0 | 0.000 |
| 10 | Jerry cans (square plastic containers with handle) | 63 | 18.139 |
| 11 | Injection gun containers | 8 | 0.441 |
| 12 | Other bottles, containers, and drums |  |  |
| 12A | Barrel liner | 58 | 2.556 |
| 12B | Other bottles, containers, and drums | 244 | 26.969 |
| 13 | Crates | 208 | 56.805 |
| 14 | Car parts | 10 | 1.166 |
| 15 | Caps / lids | 726 | 3.602 |
| 16 | Cigarette lighters | 2 | 0.020 |
| 17 | Pens | 21 | 0.065 |
| 18 | Combs / hairbrushes | 7 | 0.027 |
| 19 | Crisp / sweet packets and lolly sticks | 1 | 0.001 |
| 20 | Toys & party poppers | 44 | 1.179 |
| 21 | Cups | 0 | 0.000 |
| 22 | Cutlery / trays / straws |  |  |
| 22A | Straws | 1 | 0.000 |
| 22B | Cutlery | 5 | 0.013 |
| 22C | Plastic dishes | 1 | 0.074 |
| 22D | Other cutlery | 0 | 0.000 |
| 23 | Fertilizer / animal feed bags | 0 | 0.000 |
| 24 | Mesh vegetable bags | 0 | 0.000 |
| 25 | Gloves (typical washing up gloves) | 0 | 0.000 |
| 113 | Gloves (industrial / professional gloves) | 0 | 0.000 |
| 26 | Crab / lobster pots | 37 | 0.334 |
| 114 | Lobster and fish tags | 1 | 0.001 |
| 27 | Octopus pots | 0 | 0.000 |
| 28 | Oyster nets or mussel bags including plastic stoppers | 779 | 4.361 |
| 29 | Oyster trays (round from oyster cultures) | 2 | 0.015 |
| 30 | Plastic sheeting from mussel culture (Tahitians) | 0 | 0.000 |
| 34 | Fish boxes | 430 | 30.542 |
| 35 | Fishing line (angling) | 0 | 0.000 |
| 36 | Light sticks (tubes with fluid) | 3 | 0.050 |
| 37 | Floats / Buoys | 173 | 108.800 |
| 38 | Buckets | 183 | 21.925 |
| 39 | Strapping bands | 0 | 0.000 |
| 40 | Industrial packaging, plastic sheeting | 0 | 0.000 |
| 41 | Fiber glass | 0 | 0.000 |
| 42 | Hard hats | 2 | 0.160 |
| 43 | Shotgun cartridges | 5 | 0.016 |
| 44 | Shoes / sandals | 1 | 0.079 |
| 45 | Foam sponge | 0 | 0.000 |
| 117 | Unidentifiable fragments |  |  |
| 117A | <0.5mm | 0 | 0.265 |
| 117B | 0.5-1.5 mm | 0 | 0.772 |
| 117C | 1.5-5mm | 0 | 4.605 |
| 117D | 0.5-1.5cm | 0 | 12.898 |
| 117E | 1.5-5cm | 0 | 14.351 |
| 117F | 5-50cm | 1964 | 40.711 |
| 117G | >50cm | 35 | 104.335 |
| 48 | Other plastic / polystyrene items |  |  |
| 48A | Inner tube for wrapping | 0 | 0.000 |
| 48B | Pipes / tubes | 62 | 4.496 |
| 48C | Cylinder for fishing net repair rope | 0 | 0.000 |
| 48D | Conveyor belt items vessel | 13 | 0.256 |
| 48E | Styrofoam small (<5 cm) | 10 | 0.002 |
| 48F | Styrofoam large (>5 cm) | 8 | 0.074 |
| 48G | Detonation chord | 3 | 0.003 |
| 48H | Electrical wire | 62 | 0.098 |
| 48I | Plastic cleaning brush | 5 | 0.230 |
| 48J | Melted / burned | 182 | 7.391 |
| 48K | Baskets | 55 | 20.206 |
| 48L | Eel traps | 310 | 6.968 |
| 48M | Other plastic items | 121 | 44.613 |
|  | Sanitary waste |  |  |
| 97 | Condoms | 0 | 0.000 |
| 98 | Cotton bud sticks | 1 | 0.001 |
| 99 | Sanitary towels / panty liners / backing strips | 0 | 0.000 |
| 100 | Tampons and tampon applicators | 0 | 0.000 |
| 101 | Toilet fresheners | 4 | 0.016 |
| 102 | Other sanitary items |  |  |
| 102A | Toothpaste | 4 | 0.007 |
| 102B | Toothbrush | 56 | 0.317 |
| 102C | Ear plug | 0 | 0.000 |
| 102D | Other sanitary items | 0 | 0.000 |
|  | Medical waste |  |  |
| 103 | Containers / tubes | 0 | 0.000 |
| 104 | Syringes | 1 | 0.003 |
| 105 | Other medical items (swabs, bandaging etc.) | 2 | 0.012 |
|  |  |  |  |
|  | **Rubber** | **4** | **0.077** |
| 49 | Balloons, including plastic valves, ribbons, strings etc. | 0 | 0.000 |
| 50 | Boots | 0 | 0.000 |
| 52 | Tires and belts | 0 | 0.000 |
| 53 | Other rubber pieces | 4 | 0.077 |
|  |  |  |  |
|  | **Cloth** | **3** | **0.091** |
| 54 | Clothing | 0 | 0.000 |
| 55 | Furnishing | 0 | 0.000 |
| 56 | Sacking | 0 | 0.000 |
| 57 | Shoes (leather) | 0 | 0.000 |
| 59 | Other textiles | 3 | 0.091 |
|  |  |  |  |
|  | **Paper / Cardboard** | **5** | **0.003** |
| 60 | Bags | 0 | 0.000 |
| 61 | Cardboard | 0 | 0.000 |
| 118 | Cartons e.g., tetrapak (milk) | 0 | 0.000 |
| 62 | Cartons e.g., tetrapak (other) | 0 | 0.000 |
| 63 | Cigarette packets | 0 | 0.000 |
| 64 | Cigarette butts | 4 | 0.002 |
| 65 | Cups | 0 | 0.000 |
| 66 | Newspapers & magazines | 0 | 0.000 |
| 67 | Other paper items | 1 | 0.001 |
|  |  |  |  |
|  | **Wood (machined)** | **2** | **0.008** |
| 68 | Corks | 0 | 0.000 |
| 69 | Pallets | 0 | 0.000 |
| 70 | Crates | 0 | 0.000 |
| 71 | Crab / lobster pots | 0 | 0.000 |
| 119 | Fish boxes | 0 | 0.000 |
| 72 | Ice lolly sticks / chip forks | 0 | 0.000 |
| 73 | Paint brushes | 0 | 0.000 |
| 74 | Other wood < 50 cm | 2 | 0.008 |
| 75 | Other wood > 50 cm | 0 | 0.000 |
|  |  |  |  |
|  | **Metal** | **11** | **53.671** |
| 76 | Aerosol / Spray cans | 0 | 0.000 |
| 77 | Bottle caps | 0 | 0.000 |
| 78 | Drink cans | 0 | 0.000 |
| 120 | Disposable BBQ’s | 0 | 0.000 |
| 79 | Electric appliances | 0 | 0.000 |
| 80 | Fishing weights | 0 | 0.000 |
| 81 | Foil wrappers | 2 | 0.001 |
| 82 | Food cans | 0 | 0.000 |
| 83 | Industrial scrap | 0 | 0.000 |
| 84 | Oil drums | 0 | 0.000 |
| 86 | Paint tins | 0 | 0.000 |
| 87 | Lobster / crab pots and tops | 0 | 0.000 |
| 88 | Wire, wire mesh, barbed wire | 0 | 0.000 |
| 89 | Other metal pieces < 50 cm | 8 | 0.420 |
| 90 | Other metal pieces > 50 cm | 1 | 53.250 |
|  |  |  |  |
|  | **Glass** | **14** | **4.700** |
| 91 | Bottles | 9 | 3.110 |
| 92 | Light bulbs / tubes | 0 | 0.000 |
| 93 | Other glass items |  |  |
| 93A | Japanese glass buoy | 2 | 0.240 |
| 93B | Other glass items | 3 | 1.350 |
|  |  |  |  |
|  | **Ceramics** | **1** | **0.539** |
| 94 | Construction material (e.g., tiles) | 0 | 0.000 |
| 95 | Octopus pots | 0 | 0.000 |
| 96 | Other ceramic / pottery items | 1 | 0.539 |
|  |  |  |  |
|  | **Other** | **5** | **0.044** |
| 108 | Paraffin or wax pieces 0 - 1 cm | 0 | 0.000 |
| 109 | Paraffin or wax pieces 1 - 10 cm | 5 | 0.044 |
| 110 | Paraffin or wax pieces > 10 cm | 0 | 0.000 |
| 111 | Other pollutants | 0 | 0.000 |
| 121 | Bagged dog faeces | 0 | 0.000 |
|  |  |  |  |
| **Total** | **Entire hard debris fraction** | **6093** | **605.931** |

**Table S3|** Exemplary photos of hard plastic items collected from the NPGP during The Ocean Cleanup’s System 001/B operations and associated item categories [OSPAR ID]. Table S1 provides a complete list of item categories considered in this study.

| **Fishing and aquaculture gear** | | |
| --- | --- | --- |
| Crab / lobster pots [26] 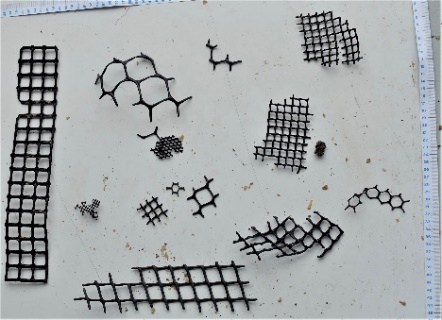 | Lobster and fish tags [114] 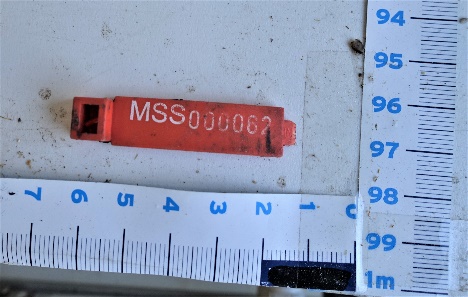 | Oyster nets, bags, spacers [28] 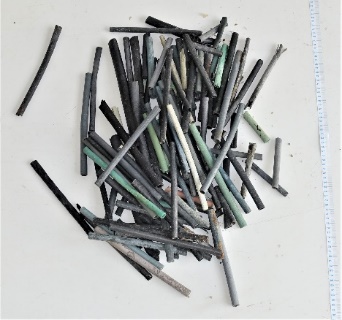 |
| Oyster trays [29] 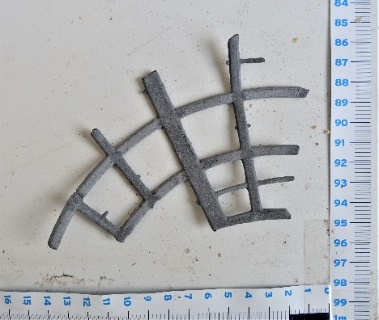 | Fish boxes [34]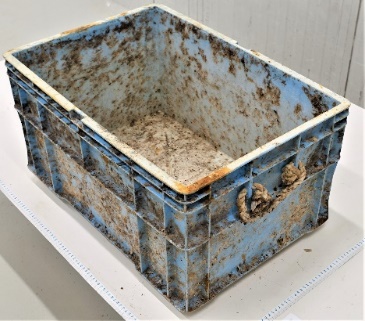 | Light sticks [36]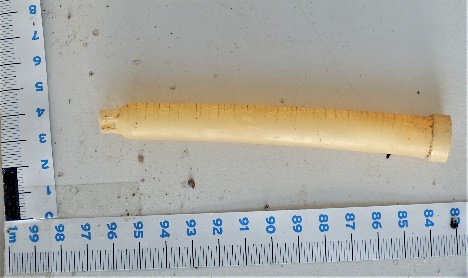 |
|  | Eel traps [48L]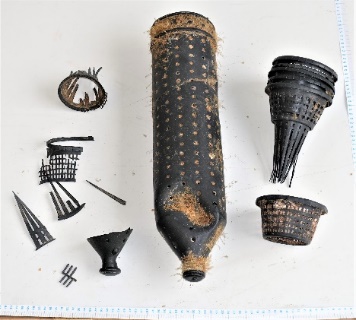 |  |
|  |  |  |
| **Floats / Buoys** [37] | | |
|  | 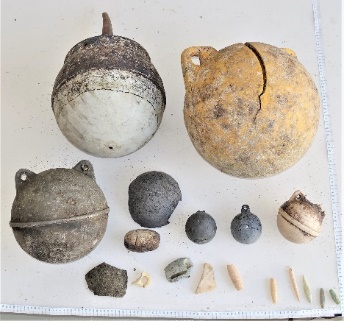 |  |
|  |  |  |
| **Crates** [13] | | |
|  | 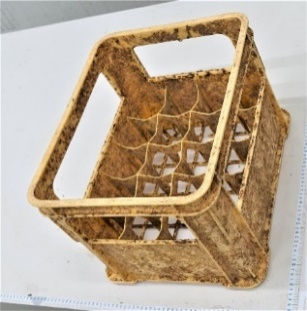 |  |
|  |  |  |
| **Buckets** [38] | | |
|  | 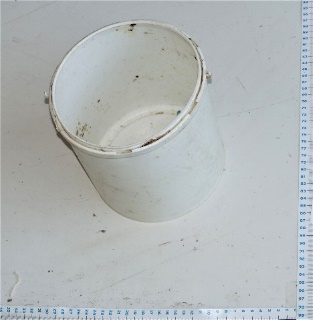 |  |
|  |  |  |
| **Food / Drinks** | | |
| Bottles, containers, and drums [4]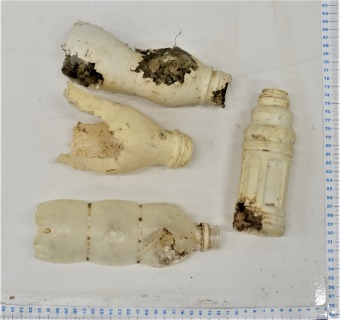 | Food containers [6]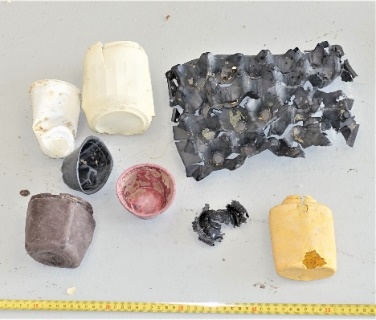 | Caps / lids [15]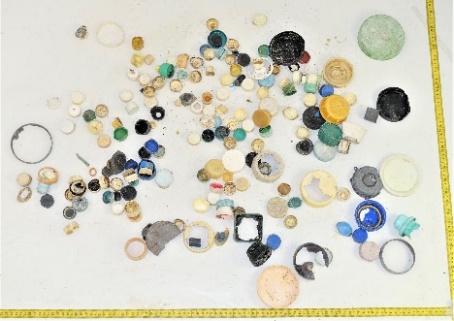 |
|  | 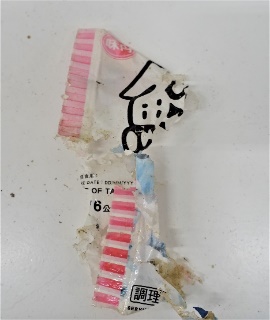Crisp / sweet packets and lolly sticks [19] |  |
|  |  |  |
| **Household items** | | |
| 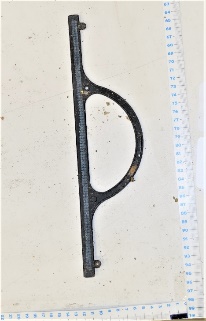Plastic bag ends [112] | 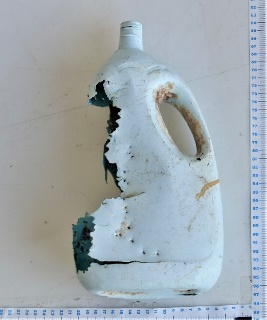Cleaner (bottles, containers, and drums) [5] | Cosmetics (e.g., sun lotion, shampoo, shower gel, deodorant) [7]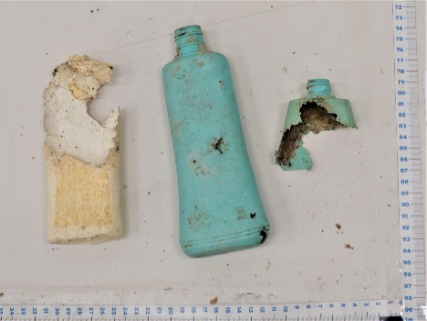 |
| Engine oil containers and drums [8]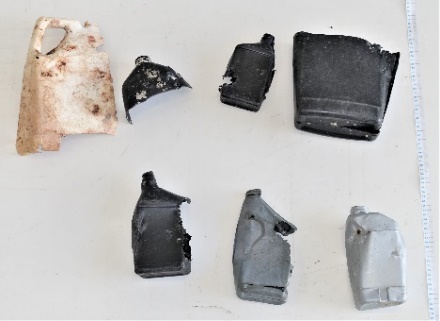 | 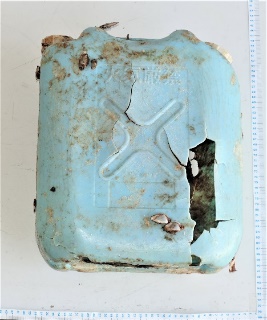Jerry cans [10] | 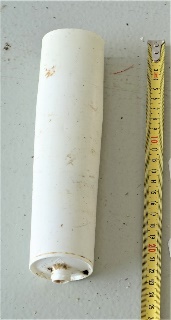Injection gun containers [11] |
| Other bottles, containers and drums [12]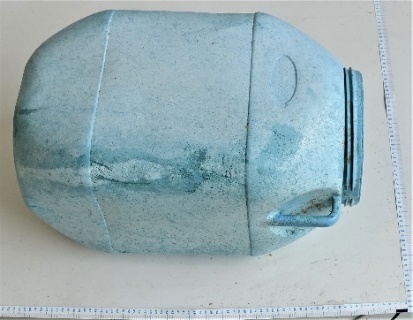 | Car parts [14]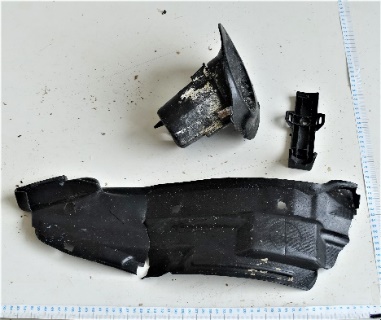 | Cigarette lighters [16] 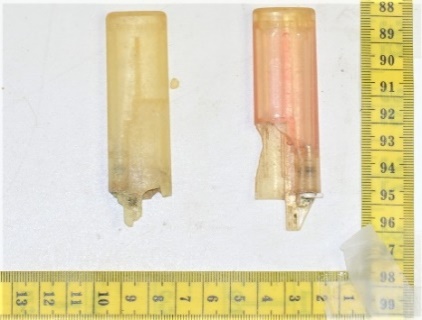 |
| 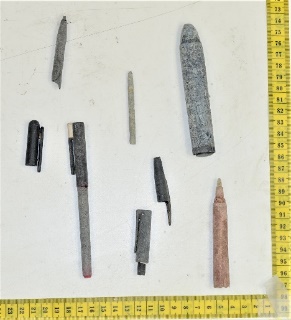Pens [17] | Combs / hairbrushes [18] 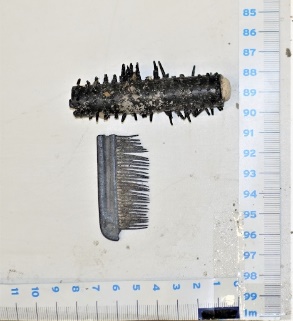 | Toys & party poppers [20]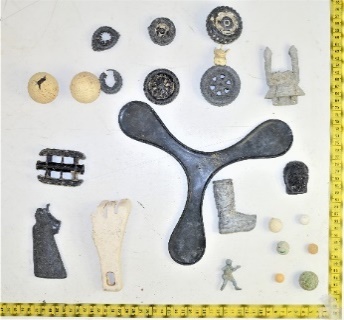 |
| Cutlery / trays / straws [22]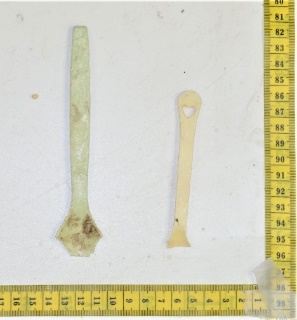 | Hard hats [42]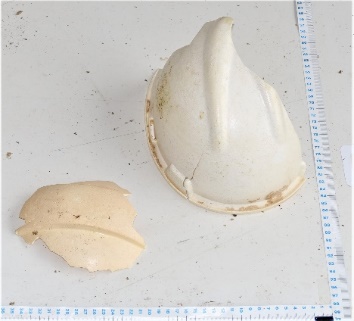 | Shotgun cartridges [43]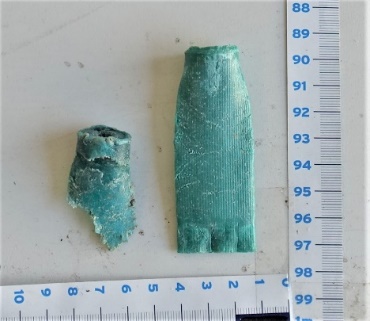 |
| Shoes / sandals [44] 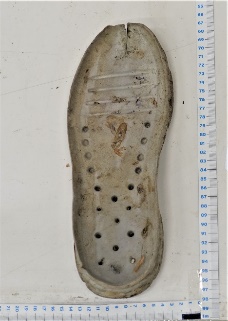 | Sanitary waste [98, 101-102]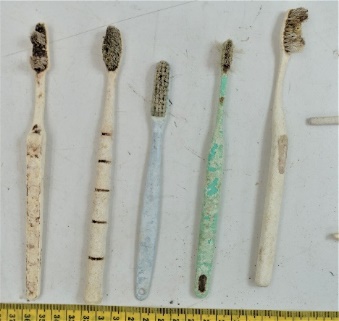 | Medical waste [104-105] 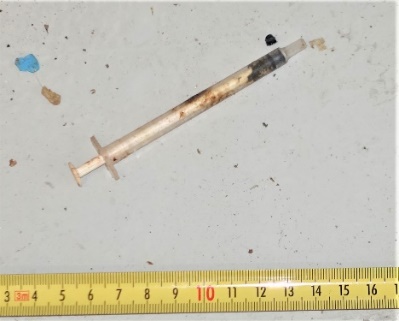 |
| Pipes / tubes [48B]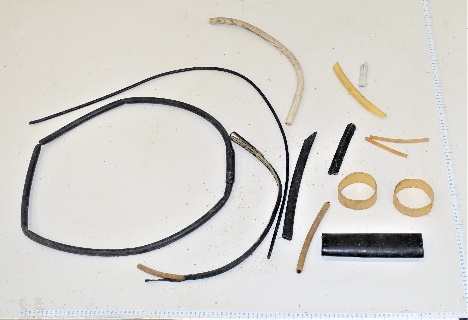 | Electrical wire [48H]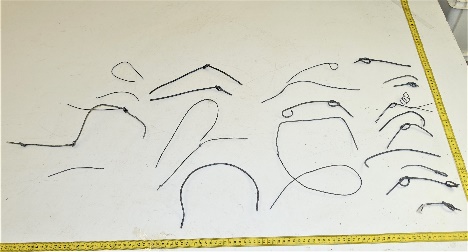 | 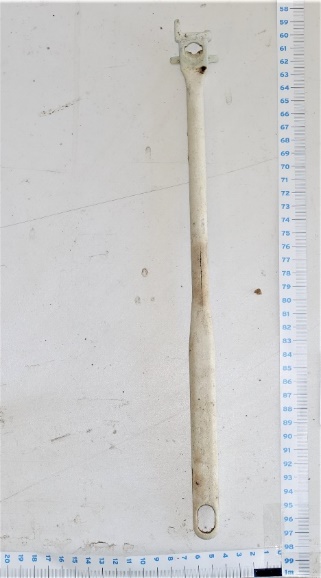Plastic cleaning brush [48I] |
|  | Baskets [48K]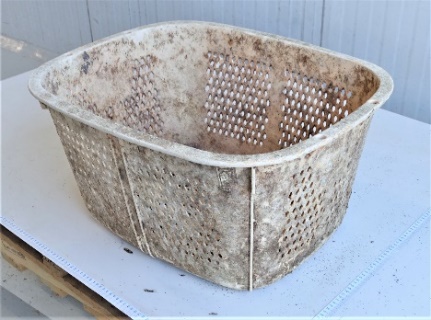 |  |
|  | | |
| **Other** | | |
| Conveyor belt items vessel [48D]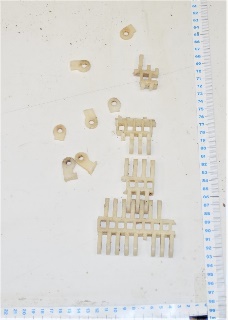 | Detonation chord [48G] 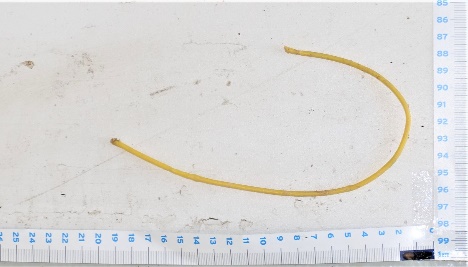 | Melted / burnt [48J] 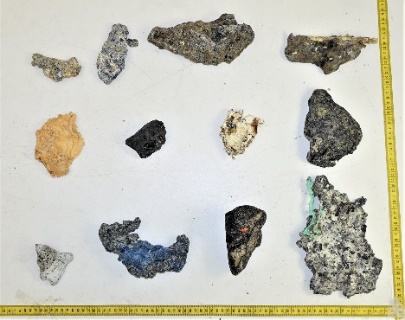 |
|  | Other plastic items [48M] 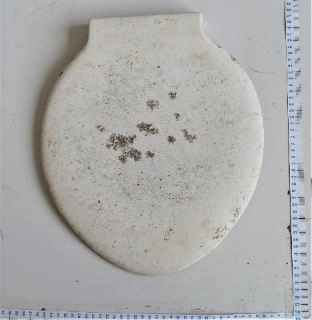 |  |
|  | | |
| **Fragments** | | |
| 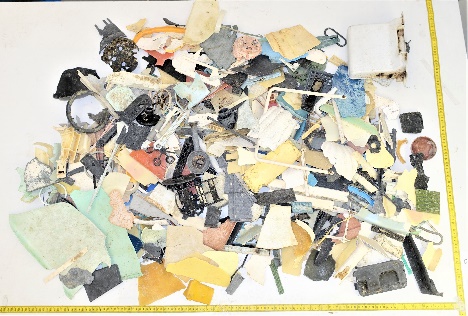Unidentifiable fragments (5-50 cm) [46-47F] | 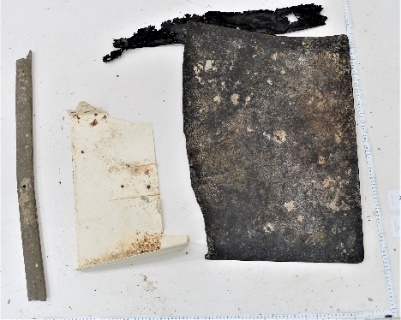Unidentifiable fragments (>50 cm) [46-47G] | 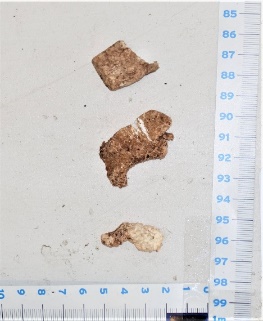Styrofoam small (<5 cm) [48E] |
|  | 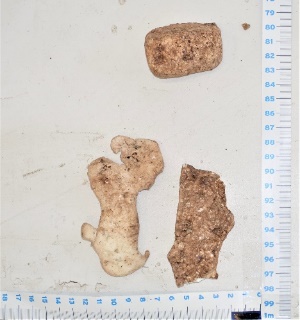Styrofoam large (>5 cm) [48F] |  |

**Table S4 |** Modelled source scenarios for global Lagrangian dispersal simulations for ocean plastics.

| **Source** | **Reference** | **# particles** | **Definition** |
| --- | --- | --- | --- |
| Rivers | Meijer et al. (2021)^3^ | 7.9*10^6^ | One model particle is released for every tonne of plastic emissions at modelled river mouth locations. The baseline is for 2015, inputs from 2013 to 2019 are scaled with annual global plastic production (Supplementary Figure S2). Particles carry the country of origin corresponding to river mouth location (i.e., upstream countries are not recorded). |
| Rivers | Borrelle et al. (2020)^4^ | 7.9*10^6^ | Same as above but model particles carry a mass converted for difference in national mismanaged plastic waste (MPW) generation between Meijer et al. (2021) and Borrelle et al. (2020). |
| Rivers | Chen et al. (2020)^5^ | 7.9*10^6^ | Same as above but model particles carry a mass converted for difference in national mismanaged plastic waste (MPW) generation between Meijer et al. (2021) and Chen et al. (2020). |
| Fishing | Kroodsma et al (2018)^6^ | 10*10^6^ | One model particle is released for every space-borne observation of fishing effort (>15 mins) in 0.1 x 0.1-degree from 2013 to 2019. Particles carry fishing effort in hours, type of gear and country flag. |

**Table S5 |** Identified global fishing effort per country and per gear of interest between 2013 and 2019^6^.

|  | **Japan** | **China** | **Korea** | **USA** | **Taiwan** | **Russia** | **Others** | **World** |
| --- | --- | --- | --- | --- | --- | --- | --- | --- |
| **Identified fishing effort globally (2013-2019, for AIS equipped vessels, in million hours)** | | | | | | | | |
| *Trawlers* | 0.4 | 50.3 | 0.9 | 3.8 | 0.8 | 7.1 | 62.9 | 126.1 |
| *Fixed gear* | 0.6 | 17.7 | 2.5 | 1.4 | 0.8 | 2.0 | 14.5 | 39.5 |
| *Drifting longline* | 6.2 | 7.5 | 3.8 | 1.3 | 12.4 | 0.0 | 9.8 | 41.1 |
| *Others / unidentified* | 0.9 | 21.1 | 2.2 | 1.7 | 1.0 | 1.0 | 15.9 | 43.9 |
| ***Total*** | **8.1** | **96.7** | **9.4** | **8.1** | **15.0** | **10.2** | **103.2** | **250.7** |

**Table S6 |** Languages identified on hard plastic objects collected from the North Pacific Garbage Patch in 2019.

| **Language** | **[#]** | **[%]** |
| --- | --- | --- |
| Chinese | 69 | 34.3 |
| Japanese | 66 | 32.8 |
| English | 35 | 17.4 |
| Korean | 21 | 10.4 |
| Chinese or Japanese | 5 | 2.5 |
| Other | 5 | 2.5 |
| *Spanish* | *2* | *1.0* |
| *Russian* | *1* | *0.5* |
| *Bahasa* | *1* | *0.5* |
| *Thai* | *1* | *0.5* |
| **Total** | **201** | **100.0** |

**Table S7 |** Origin identified on hard plastic objects collected from the North Pacific Garbage Patch in 2019. Note that the origin was identified based on a combination of languages, company logos, as well as specific text revealing clues of origin (such as an address, company name, vessel name, etc.).

| **Origin** | **Total** | | **Fishing gear ^a^** | **Others ^b^** |
| --- | --- | --- | --- | --- |
|  | **[#]** | **[%]** | **[#]** | **[#]** |
| Japan | 78 | 33.6 | 21 | 57 |
| China | 75 | 32.3 | 16 | 59 |
| Korea | 23 | 9.9 | 5 | 18 |
| USA | 15 | 6.5 | 5 | 10 |
| Taiwan | 13 | 5.6 | 2 | 11 |
| Canada | 11 | 4.7 | 1 | 10 |
| Other | 17 | 7.3 | 2 | 15 |
| *France* | 3 | 1.3 | 0 | 3 |
| *Indonesia* | 2 | 0.9 | 0 | 2 |
| *Mexico* | 2 | 0.9 | 0 | 2 |
| *Vietnam* | 2 | 0.9 | 0 | 2 |
| *Belgium* | 1 | 0.4 | 1 | 0 |
| *Colombia* | 1 | 0.4 | 0 | 1 |
| *Germany* | 1 | 0.4 | 0 | 1 |
| *Netherlands* | 1 | 0.4 | 0 | 1 |
| *Philippines* | 1 | 0.4 | 0 | 1 |
| *Russia* | 1 | 0.4 | 1 | 0 |
| *Thailand* | 1 | 0.4 | 0 | 1 |
| *UK* | 1 | 0.4 | 0 | 1 |
| **Total** | **232** | **100.0 %** | **52** | **180** |

**^a^** Items from the following OSPAR categories were assumed to likely originate from fishing activities: Crab / lobster pots [26], Oyster nets, bags, spacers [28], Oyster trays [29], Fish boxes [34], Light sticks [36], Float / buoys [37], Buckets [38], Lobster and fish tags [114], Eel traps [48L]; **^b^** Includes items from all other OSPAR categories except the categories allocated to **^a^**. This category includes household and food / drinks items such as containers and bottle caps for which it is difficult to determine whether they were discarded at sea or on land.

**Table S8 |** Global plastic production in million tonnes (Mt) for each decade and corresponding % contribution to the total global plastic production since 1950. Values for the years 1950 – 2015 are based on Geyer et al. (2017)^1^, while values for the years 2016 – 2019 were estimated by extrapolating the exponential trend between the years 1980 and 2015 (see Figure S2).

| **Years** | **Global plastic production [Mt]** | **Total % since 1950** |
| --- | --- | --- |
| 1950 – 1959 | 39 | 0.4 |
| 1960 – 1969 | 175 | 1.8 |
| 1979 – 1979 | 514 | 5.4 |
| 1980 – 1989 | 895 | 9.4 |
| 1990 – 1999 | 1558 | 16.5 |
| 2000 – 2009 | 2566 | 27.1 |
| 2010 – 2019 | 3724 | 39.3 |
| **Total** | **9471** | **100.0** |

**Table S9 |** Fishing gear categories considered in this study with global effort in hours and contribution to NPGP under different Lagrangian dispersal modelling scenarios.

| **Gear category** | **Global fishing effort from AIS equipped vessels** ^6^  **(2013-2019, in hours)** | **Contribution from model particles detected in NPGP** | | |
| --- | --- | --- | --- | --- |
|  |  | **No beaching** | **Low beaching** | **High beaching** |
| Drifting longlines | 41,104,456 | 14% | 22% | 35% |
| Seiners (seines, purse seines, tuna purse seines) | 9,146,594 | 2% | 2% | 2% |
| Trawlers | 126,140,598 | 48% | 42% | 31% |
| Pole and Line | 2,288,918 | 1% | 2% | 3% |
| Trollers | 243,992 | 0% | 0% | 0% |
| Fixed gear (set gillnet, set longlines, traps/pots) | 39,513,493 | 18% | 14% | 9% |
| Dredge fishing | 2,086,820 | 0% | 0% | 0% |
| Squid jigger | 4,883,495 | 1% | 2% | 3% |
| Unknown | 25,283,313 | 16% | 16% | 15% |

**Table S10 |** Comparison between identified origins of hard plastic items > 5 cm collected from the NPGP in 2015^3^ and in 2019 (this study) against modelled contribution of countries for different river input scenarios (expressed in tonnes)^3–5^ and fishing effort scenarios (expressed in hours of fishing activity)^6^. Results are reported for different characteristic beaching time scales and showing the relative impact of coastlines on the fate of plastic pollution from different sources. Coefficients of determination R^2^ and R^2^* between model predictions and observations are reported for regressions made respectively with and without the contribution of Japan.

| **Observations** | **Japan** | **China** | **Korea** | **USA** | **Taiwan** | **Russia** | **Others** | **Total** |  |  |
| --- | --- | --- | --- | --- | --- | --- | --- | --- | --- | --- |
| # plastic items 2015 | 124 | 114 | 64 | 16 | 5 | 1 | 25 | 349 |  |  |
| # plastic items 2019 | 78 | 75 | 23 | 15 | 13 | 1 | 27 | 232 |  |  |
| **Total** | **202** | **189** | **87** | **31** | **18** | **2** | **52** | 581 |  |  |
| **%** | **35%** | **33%** | **15%** | **5%** | **3%** | **0%** | **9%** |  |  |  |
| **Modelled river sources contributing to NPGP (2013-2019, in tonnes)** | | | | | | | | | **R^2^** | **R^2^*** |
| - no beaching (τ_beach_ = ∞) | | | | | | | | | | |
| *Meijer et al. 2021* ^3^ | 697 | 2,162 | 31 | 164 | 124 | 51 | 19,192 | 22,421 | ***0.01*** | ***0.00*** |
| *Borrelle et al. 2020* ^4^ | 34,762 | 2,724 | 634 | 1,146 | 124 | 3,101 | 21,585 | 64,076 | ***0.27*** | ***0.00*** |
| *Chen et al. 2020* ^5^ | 4,374 | 279 | 629 | 2,145 | 2,005 | 682 | 6,972 | 17,087 | ***0.00*** | ***0.07*** |
| - low beaching scenario (τ_beach_ = 24 days) | | | | | | | | | | |
| *Meijer et al. 2021* ^3^ | 314 | 745 | 3 | 62 | 64 | 22 | 5,784 | 6,995 | ***0.00*** | ***0.00*** |
| *Borrelle et al. 2020* ^4^ | 15,653 | 939 | 61 | 435 | 64 | 1,365 | 5,774 | 24,292 | ***0.35*** | ***0.00*** |
| *Chen et al. 2020* ^5^ | 1,969 | 96 | 60 | 815 | 1,039 | 300 | 1,330 | 5,610 | ***0.04*** | ***0.26*** |
| - high beaching scenario (τ_beach_ = 2 days) | | | | | | | | | | |
| *Meijer et al. 2021* ^3^ | 53 | 112 | 0 | 24 | 7 | 5 | 443 | 644 | ***0.00*** | ***0.02*** |
| *Borrelle et al. 2020* ^4^ | 2,618 | 142 | 2 | 169 | 7 | 281 | 663 | 3,881 | ***0.36*** | ***0.02*** |
| *Chen et al. 2020* ^5^ | 329 | 15 | 2 | 316 | 106 | 62 | 129 | 958 | ***0.02*** | ***0.22*** |
| **Observed fishing effort contributing to NPGP (2013-2019, in million hours)** | | | | | | | | | | |
| - no beaching (τ_beach_ = ∞) | | | | | | | | | | |
| *Trawlers* | 0.09 | 9.45 | 0.13 | 0.87 | 0.15 | 2.09 | 0.16 | 12.93 | ***0.22*** | ***0.68*** |
| *Fixed gear* | 0.10 | 2.64 | 0.34 | 0.58 | 0.13 | 0.92 | 0.03 | 4.74 | ***0.16*** | ***0.61*** |
| *Drifting longlines* | 0.93 | 0.36 | 0.50 | 0.71 | 0.90 | 0.00 | 0.22 | 3.62 | ***0.08*** | ***0.01*** |
| *Others /Unidentified* | 0.25 | 3.90 | 0.29 | 0.27 | 0.15 | 0.39 | 0.20 | 5.45 | ***0.32*** | ***0.80*** |
| *All fishing* | 1.37 | 16.35 | 1.26 | 2.43 | 1.33 | 3.40 | 0.61 | 26.74 | ***0.26*** | ***0.71*** |
| - low beaching scenario (τ_beach_ = 24 days) | | | | | | | | | | |
| *Trawlers* | 0.07 | 4.73 | 0.04 | 0.40 | 0.07 | 1.21 | 0.10 | 6.63 | ***0.21*** | ***0.65*** |
| *Fixed gear* | 0.06 | 1.18 | 0.06 | 0.24 | 0.09 | 0.50 | 0.02 | 2.14 | ***0.13*** | ***0.51*** |
| *Drifting longlines* | 0.90 | 0.34 | 0.44 | 0.70 | 0.83 | 0.00 | 0.21 | 3.41 | ***0.08*** | ***0.01*** |
| *Others/Unidentified* | 0.23 | 2.41 | 0.11 | 0.16 | 0.13 | 0.22 | 0.16 | 3.41 | ***0.34*** | ***0.79*** |
| *All fishing* | 1.25 | 8.66 | 0.65 | 1.51 | 1.12 | 1.93 | 0.49 | 15.60 | ***0.28*** | ***0.70*** |
| - high beaching scenario (τ_beach_ = 2 days) | | | | | | | | | | |
| *Trawlers* | 0.06 | 1.86 | 0.01 | 0.19 | 0.02 | 0.58 | 0.06 | 2.77 | ***0.19*** | ***0.60*** |
| *Fixed gear* | 0.03 | 0.41 | 0.01 | 0.08 | 0.03 | 0.19 | 0.01 | 0.77 | ***0.12*** | ***0.45*** |
| *Drifting longlines* | 0.85 | 0.30 | 0.37 | 0.69 | 0.75 | 0.00 | 0.19 | 3.15 | ***0.08*** | ***0.01*** |
| *Others/Unidentified* | 0.20 | 1.49 | 0.07 | 0.11 | 0.11 | 0.14 | 0.09 | 2.21 | ***0.37*** | ***0.78*** |
| *All fishing* | 1.14 | 4.07 | 0.46 | 1.06 | 0.91 | 0.91 | 0.35 | 8.90 | ***0.36*** | ***0.67*** |

**Table S11 |** Identified production dates (n = 39), as well as associated country of origin and language on hard plastic debris collected from the North Pacific Garbage Patch in 2019.

| **Language** | **1960s** | **1970s** | **1980s** | **1990s** | **2000s** | **2010s** |
| --- | --- | --- | --- | --- | --- | --- |
| Japanese | - | 2 | 2 | 2 | 1 | - |
| English | - | - | *-* | - | 2 | 2 |
| Chinese | - | 1 | - | - | - | 1 |
| Spanish | *-* | *-* | - | - | 1 | - |
| None | 1 | - | 6 | 5 | 8 | 5 |
| **Total (%)** | **2.6** | **7.7** | **20.5** | **17.9** | **30.8** | **20.5** |
|  |  |  |  |  |  |  |
| **Origin** | **1960s** | **1970s** | **1980s** | **1990s** | **2000s** | **2010s** |
| Japan | - | 2 | 2 | 2 | 2 | - |
| USA | - | - | 1 | 1 | 2 | - |
| Taiwan | - | 1 | - | - | - | 1 |
| Belgium | - | - | - | 1 | - | - |
| China | - | - | - | 1 | - | - |
| Korea | - | - | - | - | 1 | - |
| Canada | - | - | - | - | - | 1 |
| Germany | - | - | - | - | - | 1 |
| Origin unknown | 1 | - | 5 | 2 | 7 | 5 |
| **Total (%)** | **2.6** | **7.7** | **20.5** | **17.9** | **30.8** | **20.5** |

**Supplementary References**

1. Geyer, R., Jambeck, J. R. & Law, K. L. Production, use, and fate of all plastics ever made. *Science Advances* **3**, (2017).

2. OSPAR. Guideline for monitoring marine litter on the beachs in the OSPAR Maritime Area. *OSPAR Comm.* **1**, (2010).

3. Meijer, L. J. J., van Emmerik, T., van der Ent, R., Schmidt, C. & Lebreton, L. More than 1000 rivers account for 80% of global riverine plastic emissions into the ocean. *Science Advances* **7**, (2021).

4. Borrelle, S. B. *et al.* Predicted growth in plastic waste exceeds efforts to mitigate plastic pollution. *Science* **369**, (2020).

5. Chen, D. M.-C., Bodirsky, B. L., Krueger, T., Mishra, A. & Popp, A. The world’s growing municipal solid waste: trends and impacts. *Environmental Research Letters* **15**, (2020).

6. Kroodsma, D. A. *et al.* Tracking the global footprint of fisheries. *Science* **359**, (2018).

7. Lebreton, L. *et al.* Evidence that the Great Pacific Garbage Patch is rapidly accumulating plastic. *Scientific Reports* **8**, (2018).
